# Biallelic sequence and structural variants in *RAX2* are a novel cause for autosomal recessive inherited retinal disease

**DOI:** 10.1038/s41436-018-0345-5

**Published:** 2018-10-31

**Authors:** Stijn Van de Sompele, Claire Smith, Marianthi Karali, Marta Corton, Kristof Van Schil, Frank Peelman, Timothy Cherry, Toon Rosseel, Hannah Verdin, Julien Derolez, Thalia Van Laethem, Kamron N. Khan, Martin McKibbin, Carmel Toomes, Manir Ali, Annalaura Torella, Francesco Testa, Belen Jimenez, Francesca Simonelli, Julie De Zaeytijd, Jenneke Van den Ende, Bart P. Leroy, Frauke Coppieters, Carmen Ayuso, Chris F. Inglehearn, Sandro Banfi, Elfride De Baere

**Affiliations:** 1 Center for Medical Genetics, Ghent University and Ghent University Hospital, Ghent, Belgium; 2 Section of Ophthalmology and Neuroscience, School of Medicine, University of Leeds, St James’s University Hospital, Leeds, UK; 3 Medical Genetics, Department of Precision Medicine, Università degli Studi della Campania “Luigi Vanvitelli”, Naples, Italy; 4 Telethon Institute of Genetics and Medicine, Pozzuoli, Italy; 5 Genetics Department, Instituto de Investigación Sanitaria-Fundación Jimenez Diaz University Hospital (IIS-FJD, UAM), Madrid, Spain; 6 Center of Biomedical Network Research on Rare Diseases (CIBERER), ISCIII, Madrid, Spain; 7 Department of Medical Protein Research, Faculty of Medicine and Health Sciences, Flanders Institute for Biotechnology (VIB), Ghent University, Ghent, Belgium; 8 Center for Developmental Biology and Regenerative Medicine, Seattle Children’s Research Institute, Seattle, WA USA; 9 Department of Ophthalmology, St. James’s University Hospital, Leeds, UK; 10 Eye Clinic, Multidisciplinary Department of Medical, Surgical and Dental Sciences, Università degli Studi della Campania “Luigi Vanvitelli”, Naples, Italy; 11 Department of Ophthalmology, Fundación Jimenez Diaz University Hospital, Madrid, Spain; 12 Department of Ophthalmology, Ghent University and Ghent University Hospital, Ghent, Belgium; 13 Center for Medical Genetics, Antwerp University Hospital, Antwerp, Belgium; 14 Division of Ophthalmology, The Children’s Hospital of Philadelphia, Philadelphia, PA USA

**Keywords:** *RAX2*, homeobox-containing transcription factor, loss of function, retinitis pigmentosa, novel ARRP gene

## Abstract

**Purpose:**

*RAX2* encodes a homeobox-containing transcription factor, in which four monoallelic pathogenic variants have been described in autosomal dominant cone-dominated retinal disease.

**Methods:**

Exome sequencing in a European cohort with inherited retinal disease (IRD) (*n* = 2086) was combined with protein structure modeling of RAX2 missense variants, bioinformatics analysis of deletion breakpoints, haplotyping of *RAX2* variant c.335dup, and clinical assessment of biallelic *RAX2*-positive cases and carrier family members.

**Results:**

Biallelic *RAX2* sequence and structural variants were found in five unrelated European index cases, displaying nonsyndromic autosomal recessive retinitis pigmentosa (ARRP) with an age of onset ranging from childhood to the mid-40s (average mid-30s). Protein structure modeling points to loss of function of the novel recessive missense variants and to a dominant-negative effect of the reported dominant *RAX2* alleles. Structural variants were fine-mapped to disentangle their underlying mechanisms. Haplotyping of c.335dup in two cases suggests a common ancestry.

**Conclusion:**

This study supports a role for *RAX2* as a novel disease gene for recessive IRD, broadening the mutation spectrum from sequence to structural variants and revealing a founder effect. The identification of biallelic *RAX2* pathogenic variants in five unrelated families shows that *RAX2* loss of function may be a nonnegligible cause of IRD in unsolved ARRP cases.

## INTRODUCTION

The development of the vertebrate eye is a well-coordinated multistep process regulated by the interplay of genetic networks and interactions with the extracellular environment. During early development of the vertebrate central nervous system, the eye field forms centrally within the anterior neural plate, containing all the progenitors of the neural-derived eye structures.^1^ This field is defined by the area where the expression domains of a set of eye field transcription factors (TFs) overlap. TFs such as Pax6, Rax, Six3, and Lhx2 are homeobox-containing proteins that constitute a regulatory network to specify retinal progenitor cells, giving rise to eye structures such as the neural retina and the retinal pigment epithelium (RPE).^2^ In particular, members of the retinal homeobox (*Rax*) gene family are among the earliest markers of the eye field, playing a pivotal role during vertebrate eye development. Loss of function of *RAX1* orthologues in mice,^3^ medaka,^4^ zebrafish,^5^ and *Xenopus*^6^ results in an eyeless phenotype due to failure of optic vesicle formation, indicating the essential role of *RAX1* genes in normal eye development. In contrast, *RAX2* deficient chicken,^7^ *Xenopus*,^8^ and zebrafish^9^ models demonstrate that *RAX2* is required for cell proliferation and differentiation within the retina by regulating the spatial expression of photoreceptor-specific genes in late retinogenesis.

In humans, the *RAX* paralogues appear to function in a manner similar to that observed in other species, because genetic defects in *RAX* have been associated with both anophthalmia and microphthalmia,^10^ while pathogenic variants in *RAX2*^11,12^ cause autosomal dominantly inherited retinal disease (IRD). RAX2 shares a nearly identical (93%) homeodomain with RAX, although limited homology is observed elsewhere. Its expression is mainly limited to the outer and inner nuclear layers of the retina. RAX2 functions as a transcriptional coactivator by synergistically increasing the transactivation activity of the photoreceptor-specific TFs through physical interaction with CRX.^11^

So far, only a handful of likely pathogenic *RAX2* variants (NM_032753) have been described. Wang et al. reported three heterozygous sequence variants in single patients with IRD, which they tentatively associated with central retinal degeneration.^11^ The c.409G>C p.(Gly137Arg) and c.417_422dup p.(Pro140_Gly141dup) variants were found in two patients with cone–rod dystrophy (CRD). These variants exhibit, respectively, a reduced and an increased transactivation activity in vitro in the presence of CRX and NRL, as well as a decreased interaction with CRX. The c.260G>A p.(Arg87Gln) variant was found in a patient with age-related macular degeneration (AMD), and linked to an increased transactivation function in vitro. However, because no segregation analysis was performed, the authors only raised the possibility that *RAX2* may be involved in disease pathogenesis. Yang et al. identified one additional heterozygous *RAX2* variant, c.465_475del p.(Ala156Argfs*131), in a family with autosomal dominant cone or cone–rod dystrophy.^12^

Here, we report the identification of *RAX2* as a novel disease gene mutated in autosomal recessive retinitis pigmentosa (ARRP), a rod–cone type of IRD, in five unrelated families of Belgian, British, Italian, and Spanish origin.

## MATERIALS AND METHODS

### Patients and clinical evaluation

This study was approved by local ethics committees in Belgium, Italy, Spain, and the United Kingdom (Supplementary File 1) and all participants gave informed consent. Clinical assessment consisted of recording of a medical history and of ophthalmic assessment, including fundus examination, optical coherence tomography (OCT), visual acuity measurement, Goldmann visual field testing, electroretinographic (ERG) testing following International Society for Clinical Electrophysiology of Vision (ISCEV) standards, and color vision assessment.

### Exome sequencing

Genomic DNA was extracted from blood using the DNeasy Blood & Tissue Kit (QIAGEN). For patients I, II, and V exome enrichment was performed with the SureSelectXT Human All Exon V5/V6 kit (Agilent), followed by paired-end sequencing on a HiSeq 3000 system (Illumina). For patient III, exome sequencing libraries were prepared using the SureSelectQXT Clinical Research Exome V1 kit (Agilent) and run on a NextSeq 500 system (Illumina). For patient IV, clinical exome sequencing was performed using the commercial TruSight One panel (Illumina) on a NextSeq 500 system (Illumina). Read processing and variant calling were performed using in-house developed pipelines.^13–17^ Copy-number analysis was performed using the R package ExomeDepth.^18^ Variants were confirmed and segregated by Sanger sequencing using the BigDye Terminator v3.1 kit (Life Technologies). Primer sequences are listed in Supplementary Table 1.

### Haplotype analysis of the recurrent Belgian variant c.335dup

Runs of homozygosity (ROH) were determined from exome sequencing data using the H3M2 algorithm.^19^ Additional single-nucleotide polymorphisms (SNPs) were genotyped using conventional polymerase chain reaction (PCR) and Sanger sequencing (Supplementary Table 2). Microsatellite primers were PCR amplified and products were analyzed on an ABI Prism 3730xl Genetic Analyzer (Applied Biosystems). Microsatellite genotyping calls were generated using GeneMapper v5 software (Applied Biosystems).

### Structural analysis of the RAX2 missense variants

Homology models for the RAX2 homeodomain were built using automated homology modeling with automated template searching in the Yasara software.^20^ Protein Data Bank (PDB) codes of superposed structures are 3A01_A, 3A01_B, 1FLJ_A, 2H1K_A, 1IG7_A, 5Z2T_C. Models of the RAX2/CRX/Ret-1 complex were built using the crystal structure of aristaless and clawless homeodomains bound to DNA (3A01) as template with a custom ungapped sequence alignment. The template was first adapted by mutating the DNA fragments to the Ret-1 sequence GGGGCTTAATTGGCT and its complement. Models of the RAX2/CRX/Ret-1 complex were further analyzed using molecular dynamics in Yasara with 160-ns simulations.^21^ Effects of variants on stability and interactions were predicted with FoldX, using the mutate residue command with initial RepairPDB. Structure superpositions, Ramachandran plot, and images were created in University of California–San Francisco (UCSF) Chimera.

### Fine-mapping and bioinformatics study of the deletion breakpoints

For the determination of deletion breakpoints, the High Fidelity LA Taq DNA Polymerase (Takara) was used for long range PCR. Amplicons of the proband were Sanger sequenced and aligned to the reference genome (hg19) using the University of California–Santa Cruz (UCSC) Genome Browser (http://genome.ucsc.edu/).

For the *RAX2* deletions identified in this study, a bioinformatics analysis was performed to assess the underlying mechanism, as previously described.^22^ In particular, for the breakpoint regions of the deletions we analyzed the degree of microhomology (multiple sequence alignment, ClustalW), the presence of repetitive elements (RepeatMasker track, UCSC Genome Browser), the percentage of sequence identity between repeat elements of the same class (BLAST), and the presence of sequence motifs, based on 40 previously described motifs (Fuzznuc).^23^

An integrated epigenomic profile of the *RAX2* locus in adult human retina was obtained by alignment of profiles obtained by ATAC-seq and multiple ChIP-seq (H3K4me2, H3K27ac, Crx, Otx2) experiments as described (www.biorxiv.org/content/early/2018/09/08/412361).

## RESULTS

### Clinical findings

**Fig. 1:**
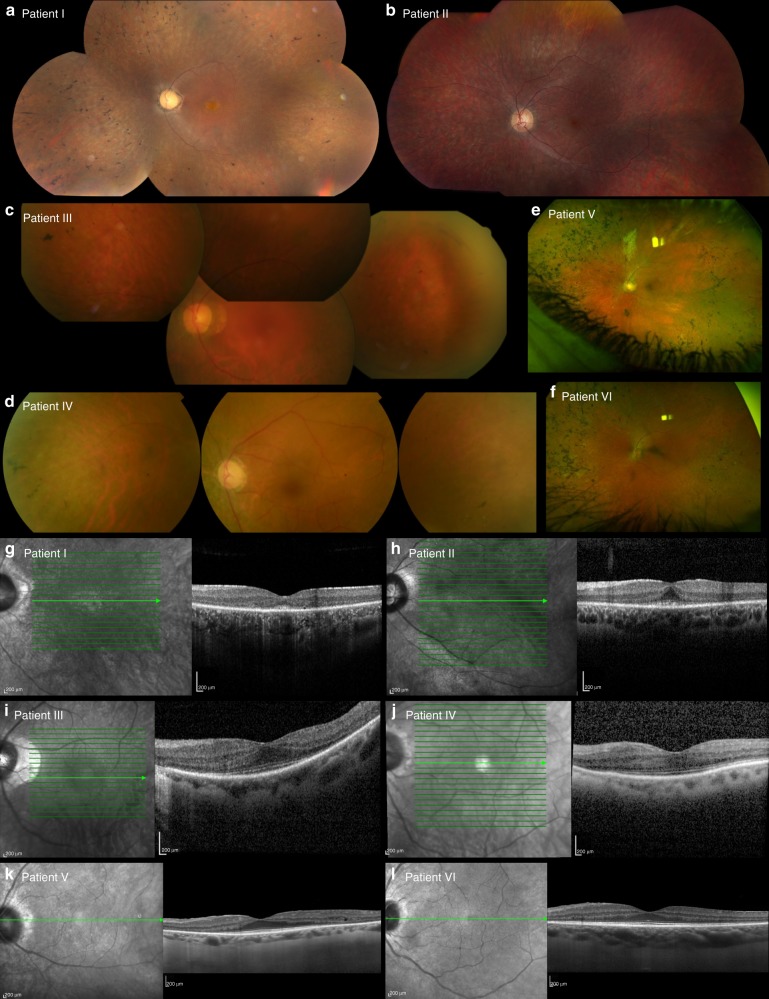
**Fundus and optical coherence tomography (OCT) imaging in patients with** ***RAX2*-associated ARRP.** **a**–**f** Fundus images and (**g**–**l**) OCT images of patients I–VI (left eye) show features characteristic of retinitis pigmentosa as described in further detail in Table 1 and Supplementary File 2. Notable features include variable degrees of pigmentation and a general preservation of foveal structure.

The families included in this study are of Belgian, British, Italian, and Spanish origin and are part of a larger IRD cohort (*n* = 2086, Supplementary Table 3) from the European Retinal Disease Consortium (ERDC, https://www.erdc.info), which aims to identify novel IRD genes and pathogenetic mechanisms. All patients included were clinically diagnosed with nonsyndromic retinitis pigmentosa (RP). Fundus and OCT imaging revealed typical features of RP (Fig. 1) and visual fields were restricted to about ten degrees in all patients. Visual acuity ranged from normal (11/10) to hand movements (1/10), with an average of 6/10. The age of onset varied from childhood to mid-40s, with an average around mid-30s. A possible consanguineous origin was reported in patients III and IV. More detailed ophthalmic characteristics are provided in Table 1 and Supplementary File 2. Fundus autofluorescence images of all patients and ERG traces of patients II, III, and IV are provided in Supplementary Figures 1 and  2 respectively.

**Table 1:** Overview of clinical findings in *RAX2*-associated ARRP

	Age/sex	Age of onset	VA (OD/OS)	Goldmann visual field	Fundus imaging	OCT	ERG	Other findings
**Patient I**	37/F	Mid-30s	7/10–7/10	Restricted to central 10°	Pale optic disc, very narrow retinal vessels, peripheral pigmentation	Foveal island with photoreceptor sparing, loss of outer retinal layers outside	-	Mixed red/green blue/yellow color vision deficiency
**Patient II**	22/M	Childhood	8/10–7/10	Restricted to central 10°	Pale optic disc, narrowed retinal vasculature, limited intraretinal pigmentation	Foveal island with photoreceptor sparing, loss of outer retinal layers outside	Nondetectable rod and cone response, flat ERG	Moderate red/green color vision deficiency
**Patient III**	58/F	Mid-30s	4/10–4/10	Restricted to central 10°	Rose-colored optic disc, narrowed retinal vessels, rare bone spicule pigmentation	Preserved retinal layers in fovea, small intraretinal cystic changes perifoveal region	Nondetectable rod response, reduced cone response	Perifoveal ring of high density and reduced autofluorescence in the periphery
**Patient IV**	61/F	Mid-40s	NA–6/10	Restricted to central 10°	Pale optic disc, narrowed retinal vasculature, bone spicule pigmentation	Preserved outer retinal layers in macula, disruption of ellipsoid band nasally from foveola	Nondetectable rod response, preservation of cone response	Glaucoma and cataract; enucleation of right eye
**Patient V**	62/F	Mid-40s	1/10–7/10	Restricted to central 10°	Pale optic disc, narrow retinal vessels, widespread bone spicule pigmentation	Preserved outer retinal structures in macula, loss of outer nuclear layer in periphery	–	Speckled hypoautofluorescence, central ring of hyperautofluorescence around the macula
**Patient VI**	52/M	Mid-40s	10/10–11/10	–	Pale optic disc, narrow retinal vessels, widespread bone spicule pigmentation	Preserved outer retinal structures in macula, loss of outer nuclear layer in periphery	–	Speckled hypoautofluorescence, central ring of hyperautofluorescence around the macula

Patient I is a 37-year-old Belgian female with simplex RP. She was diagnosed at the age of 34 and suffers from night blindness and a reduced visual field. Patient II was diagnosed with simplex RP in his mid-teens. Symptoms of night blindness started around the age of five. Patient III is a 58-year-old female patient of Italian origin whose family originates from a small village in southern Italy. She was diagnosed with simplex RP at the age of 35 years and reported onset of night blindness at the age of 51 years. The medical history did not reveal any systemic conditions and family history was negative. Patient IV is a 61-year-old Spanish female with a history of glaucoma and cataract, which resulted in the enucleation of the right eye in 2009. She was diagnosed with simplex RP at the age of 48 years, suffering from night blindness, constriction of the visual field, and loss of visual acuity. Her family originates from an endogamic village in the north of Spain. Patient V, a 60-year-old British female and her brother (patient VI) showed mild symptoms of RP starting from their mid-40s. Their parents are unrelated. Fundus autofluorescence images of all patients are provided in Supplementary Figure 1. ERG profiles of patients II, III, and IV are provided in Supplementary Figure 2.

*ERG* electroretinography, *OCT* optical coherence tomography, *OD* *oculus dexter*, *OS* *oculus sinister*, *VA* visual acuity.

### Genetic findings

**Fig. 2:**
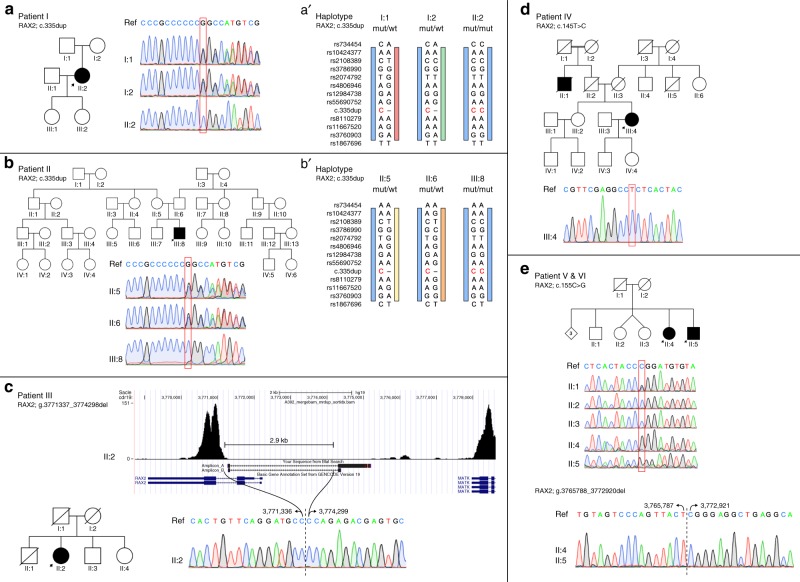
**Overview of identified** ***RAX2*** **variants in five families with ARRP.** **a** Pedigree of patient I, in which *RAX2* variant c.335dup p.(Ala113Glyfs*178) was found. Sanger sequencing traces indicate homozygosity in the patient (II:2) and heterozygosity in the unaffected parents (I:1, I:2). **b** Pedigree of patient II, in which *RAX2* variant c.335dup p.(Ala113Glyfs*178) was found. Sanger sequencing traces indicate homozygosity in the patient (III:8) and heterozygosity in the unaffected parents (II:5, II:6). **c** Pedigree of patient III, in which homozygous *RAX2* deletion g.3771337_3774298del was found. The absence of reads mapped to the first two exons of *RAX2* is visible on the exome sequencing coverage plot. The sequence of the junction polymerase chain reaction (PCR) product spanning the 2.9 kb deletion is depicted below. **d** Pedigree of patient IV, in which *RAX2* variant c.145T>C, p.(Ser49Pro) was found. Sanger sequencing traces indicate homozygosity in the patient (III:4). **e** Pedigree of patient V and VI, in which *RAX2* variants c.155C>G p.(Pro52Arg) and g.3765788_3772920del were found. Sanger sequencing traces indicate hemizygosity in the patients (II:4, II:5). One of the three analyzed healthy siblings is heterozygous for the missense variant (II:1), while the other two did not carry this variant (II:2, II:3). The sequence of the junction PCR product spanning the 7.1 kb deletion is depicted below. *RAX2* variant nomenclature uses numbering with the A of the initiation codon ATG as +1 based on transcript NM_032753. (**a**’, **b**’) Haplotype analysis of the c.335dup *RAX2* variant. Genotyping of patients I, II, and both of their unaffected parents, using 24 intragenic single-nucleotide polymorphisms (SNPs) and 3 microsatellites located in a region of 1.4 Mb around the variant. The c.335dup *RAX2* variant is indicated in red. The blue bar represents the common disease haplotype, from SNP rs10424377 to SNP rs3760903. The maximal common region in the two families spans 447.3 kb. *mut* mutant, *wt* wild type.

In five apparently unrelated families of Belgian (patients I–II), Italian (patient III), Spanish (patient IV), and British (patients V–VI) origin, biallelic novel *RAX2* variants were identified (5/2086, 0.24%; estimate of 1000 RP cases: 5/1000, 0.50%). A summary of the molecular genetic findings can be found in Fig. 2 and Table 2. An overview of the exome sequencing variant filtering for each patient can be found in Supplementary File 3.

**Table 2:** Overview of molecular findings in *RAX2*-associated autosomal recessive retinitis pigmentosa (ARRP)

	Genotype				Population frequency			Prediction of pathogenicity						
	g.notation (hg19)	c.notation	p.notation	Zygosity	gnomAD	ExAC	dbSNP	Variant class (ACMG)	Polyphen2	SIFT	Mutation Taster	CADD	Align GVGD	Grantham score
**Patient I**	chr19:g.3770839dup	c.335dup	p.Ala113Glyfs*178	Homozygous	8.40E-06	–	–	Pathogenic (class 5)	–	–	–	–	–	–
**Patient II**	chr19:g.3770839dup	c.335dup	p.Ala113Glyfs*178	Homozygous	8.40E-06	–	–	Pathogenic (class 5)	–	–	–	–	–	–
**Patient III**	chr19:g.3771337_3774298del	–	–	Homozygous	–	–	–	Pathogenic (class 5)	–	–	–	–	–	–
**Patient IV**	chr19:g.3771596A>G	c.145T>C	p.Ser49Pro	Homozygous	–	–	–	Likely pathogenic (class 4)	Probably damaging	Deleterious	Disease causing	26.5	C65	74
**Patients V & VI**	chr19:g.3771586G>C	c.155C>G	p.Pro52Arg	Heterozygous	–	–	–	Likely pathogenic (class 4)	Probably damaging	Deleterious	Disease causing	26.9	C65	103
	chr19:g.3765788_3772920del	–	–	Heterozygous	–	–	–	Pathogenic (class 5)	–	–	–	–	–	–

Identified variants were classified according to the American College of Medical Genetics and Genomics (ACMG) standards and guidelines. The two missense variants were scored by six in silico prediction tools.

Exome sequencing in unrelated patients I and II revealed a novel frameshift variant, c.335dup p.(Ala113Glyfs*178), in homozygous state in exon 3 of *RAX2*. Except for one heterozygous entry in the European population in the Genome Aggregation Database (gnomAD, r2.0.1) (0.00084%), this variant is absent from other public databases (1000 Genomes Project, ExAC, dbSNP, HGMD) (Table 2). Because of its location in the last exon of *RAX2*, this variant is predicted to escape nonsense mediated decay (NMD), giving rise to a putative read-through protein that is 107 amino acids longer than the wild-type RAX2 protein. An overview of other biallelic variants identified by exome sequencing in patients I and II is given in Supplementary Table 4. Based on gene function and variant effect, the homozygous *RAX2* frameshift variant appears to be the most plausible pathogenic variant in relation to the retinal phenotype. The pathogenic *RAX2* variant was confirmed by Sanger sequencing and segregation was demonstrated in both parents (Fig. 2a, b). Fundus autofluorescence images of the unaffected carrier mother of patient II are provided in Supplementary Figure 3A and did not reveal any sign of retinal impairment.

The analysis of variants identified by exome sequencing in patient III did not yield any high-confidence pathogenic variants in known or candidate IRD genes (Supplementary Table 4). Hence, the presence of copy-number variants (CNVs) was evaluated by analyzing the exome sequencing coverage data. This revealed that the 5’ end of the *RAX2* gene was devoid of any reads, suggesting a homozygous deletion (Fig. 2c). Two primer pairs that anneal outside the putative deleted region were used to delineate the deletion breakpoints (Supplementary Table 1). Sanger sequencing of the junction products determined the breakpoint extremities at nucleotide level (chr19:3,771,337-3,774,298). The deleted region of 2.9 kb spans the first two *RAX2* exons, one noncoding and one coding, containing the start and subsequent 71 codons, spanning most of the homeodomain. Segregation analysis was performed in two unaffected siblings (II:3, II:4), who were found to be heterozygous for the deletion. Both II:3 and II:4 underwent full ophthalmological assessment, including fundus autofluorescence, OCT, and standard ERG. Neither of them showed any sign of visual impairment (Supplementary Figure 3B).

**Fig. 3:**
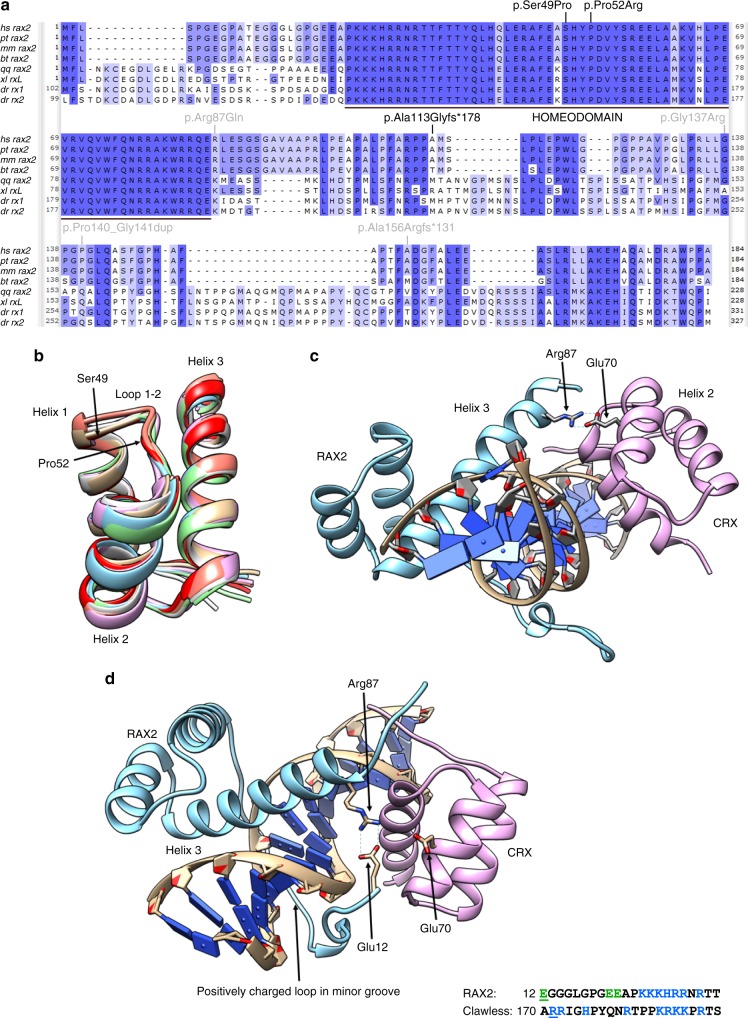
**Protein alignment with location of RAX2 variants and RAX2 structural analysis.** **a** Amino acid alignment of RAX2 orthologues. Amino acid sequences deduced from the nucleotide sequences were downloaded from the National Center for Biotechnology Information (NCBI) (https://www.ncbi.nlm.nih.gov/). The accession numbers of the sequences used in this alignment study are as follows. *Homo sapiens*: hs_rax2, NM_032753; *Pan troglodytes*: pt_rax2, NM_001081487; *Macaca mulatta*: mm_rax2, XM_001100945; *Bos taurus*: bt_rax2, NM_182653; *Gallus gallus*: gg_rax2, AF420601; *Xenopus laevis*: xl_rxL, DQ360108; *Danio rerio*: dr_rx1, AF001907; *Danio rerio*: dr_rx2, AF001908. The alignment was performed using the MUSCLE algorithm with default parameters in the Unipro UGENE v1.29.0 software. Gaps required for optimal alignment are indicated by dashes. The color intensity of the amino acids is according to percentage identity. The highly conserved RAX2 homeodomain is indicated by a black bar. The three RAX2 pathogenic sequence variants identified in this study are indicated at the amino acid level in black. The four previously reported RAX2 pathogenic variants involved in autosomal dominant cone-dominated retinal disease are indicated at the amino acid level in gray. **b** Homology modeling of the RAX2 homeodomain. Structure superposition of a homology model of the RAX2 homeodomain with six homeodomain crystal structures. The loop between helix 1 and 2, containing Ser49 and Pro52, is structurally conserved. Protein Data Bank (PDB) codes of superposed structures are 3A01_A, 3A01_B, 1FLJ_A, 2H1K_A, 1IG7_A, 5Z2T_C. **c** Model for RAX2 and CRX bound to a Ret-1 fragment. In the model, Arg87 of RAX2 interacts with Gln70 of CRX. PDB codes of structure used as template: 3A01_A. **d** Model with the N-terminal extension of RAX2. In this model, a positively charged region inserts in the minor groove. The RAX2 homeodomain and its N-terminal domain completely encircle the DNA fragment, and the ring is closed by a Glu12-Arg87 salt bridge. The sequence of the positively charged region in RAX2 and its counterpart in the clawless template are shown. RAX2 residue Glu12 and clawless site 1 residue (Arg171) are underlined. Green: negatively charged amino acids. Blue: positively charged amino acids.

Exome sequencing revealed a novel homozygous missense variant, c.145T>C p.(Ser49Pro), in the first coding exon of *RAX2* in patient IV. This variant, which is located in the second largest homozygous region of this patient with possible consanguineous origin, is absent from public databases, is predicted to be pathogenic by different computational algorithms (Table 2), and changes a highly conserved serine residue located in the homeobox domain (Fig. 3a). No additional (likely) pathogenic variants remained after filtering. The variant was confirmed by Sanger sequencing and found to be heterozygous in the unaffected sister (III:2) (Fig. 2d).

Exome sequencing in patient V revealed potentially biallelic variants in a total of five genes. An annotated list of these variants is given in Supplementary Table 4. Of these, two genes were identified as being known IRD genes, *EYS* and *RAX2*. Segregation analysis of the *EYS* variants by Sanger sequencing enabled these to be ruled out as causative for disease. The variant in *RAX2*, c.155C>G p.(Pro52Arg), appeared to be homozygous in patient V as well as in patient VI. Subsequent CNV analysis of the exome sequencing data demonstrated the presence of a heterozygous 7.1 kb deletion spanning the entire *RAX2* coding region extending to the 3’ end of the *MRPL54* gene. Sanger sequencing of the PCR junction products determined the breakpoint extremities at nucleotide level (chr19:3,765,788-3,772,920). This led to the conclusion that c.155C>G p.(Pro52Arg) is hemizygous. Segregation analysis revealed heterozygosity of the missense variant in one unaffected sibling (II:1) and absence in two others (II:2, II:3) (Fig. 2e). The deletion was present in the affected siblings, patient V and VI, while neither of the unaffected siblings was a carrier of the deletion (Supplementary Figure 4). The novel missense variant p.(Pro52Arg) changes a highly conserved proline residue located in the homeobox domain (Fig. 3a), is absent from public databases, and is predicted to be pathogenic by several computational algorithms (Table 2).

### Haplotype analysis of the recurrent Belgian variant c.335dup

Because the same *RAX2* variant, c.335dup p.(Ala113Glyfs*178), was identified in patients I and II, both of Belgian origin, haplotype analysis was performed in these two patients and four carriers (parents of the patients) to assess a common ancestry. Genotyping of 24 SNPs and three microsatellites distributed over a 1.3 Mb region showed a common disease haplotype in the two RP patients and four unaffected carriers of 447.3 kb with 6.2 kb and 13.7 kb border regions (Fig. 2a’, b’) (Supplementary Figure 5). These findings are consistent with a founder effect.

### Protein structure modeling of the RAX2 missense variants

Several homology models were built for the RAX2 homeodomain, based on different homeodomain template structures. In all models, residues that are in proximity to DNA are identical between RAX and RAX2, indicating that they bind the same response elements. Residues Ser49 and Pro52 are both part of the structurally conserved extended loop between helix 1 and 2 of the homeodomain (Fig. 3b): Ser49 adapts a backbone conformation that is not allowed for proline (Supplementary Figure 6A) and the Pro52 proline ring inserts into the hydrophobic core of the homeodomain three-helix bundle (Supplementary Figure 6B). Pfam alignment of homeodomains confirms that only a proline or hydrophobic residue is allowed at position 52. Both p.(Ser49Pro) and p.(Pro52Arg) variants are therefore expected to strongly affect the folding and/or stability of the RAX2 protein, as supported by FoldX stability computations in the homology models, resulting in a loss of function.^24^

The aristaless and clawless homeodomains used to build the model for the RAX2/CRX/Ret-1 complex bind at opposite sides of a DNA fragment and directly interact with each other via two important salt bridges.^25^ RAX2 and CRX interact with each other in analogous fashion while bound to the photoreceptor conserved element-1 (PCE-1/Ret-1) ^11,26^. In this complex, the Arg87 residue located at the C-terminal end of the RAX2 homeodomain helix 3 makes a stable salt bridge with Glu70 of the CRX homeodomain (Fig. 3c). The subsequent substitution of arginine by glutamine, p.(Arg87Gln), found in a patient with dominant AMD,^11^ disrupts this salt bridge. In addition, homology modeling suggests that the N-terminal extension of RAX2 encircles the DNA strand and binds in the minor groove via a positively charged binding motif that strongly resembles the motif in clawless (Fig. 3d). Unlike in clawless, the N-terminal RAX2 extension contains three negative charges, which are in close proximity to the positively charged Arg87, suggesting a role of this residue in binding its own N-terminus encircling the DNA (Fig. 3d). Moreover, FoldX analysis does not indicate any effect of the p.(Arg87Gln) variant on protein stability or DNA-binding of the isolated RAX2 homeobox domain. We therefore speculate on a potential role of Arg87 in homeodomain–homeodomain interactions at response elements, which is affected by p.(Arg87Gln), suggestive for a dominant-negative effect. This role is not necessarily limited to RAX2/CRX interaction but may even apply to RAX2 homodimer formation.

### Bioinformatics findings of the *RAX2* CNVs

To assess the underlying mechanisms of the *RAX2* CNVs identified in patients III, V, and VI, bioinformatic analyses were performed on the breakpoint regions of these deletions. A summary of the findings is given in Supplementary Table 5, while the presence of microhomology, repeats, and sequence motifs is visualized in Supplementary Figure 7. Based on the results of these extensive analyses, the partial *RAX2* deletion identified in patient III (chr19:3,771,337-3,774,298) may be caused either by nonhomologous end-joining (<5-bp microhomology) or by a replicative-based repair mechanism. The absence of an information scar, typical of nonhomologous end-joining (NHEJ), favors the latter hypothesis. Similarly, the presence of 4-bp microhomology at the junction of the complete *RAX2* deletion identified in patients V and VI (chr19:3,765,788-3,772,920) points to NHEJ or a replicative-based repair mechanism. As the breakpoints of the complete deletion both overlap with an *Alu*-repeat, *Alu*-*Alu*-mediated nonallelic homologous recombination (NAHR) cannot be ruled out. However, these repeats probably do not share sufficient homology to be used as substrates for NAHR.^27^ An assessment of the presence of putative *cis*-regulatory elements in the *RAX2* CNVs can be found in Supplementary Figure 8. Deletion g.3771337_3774298del overlaps with the *RAX2* promoter and deletion g.3765788_3772920del overlaps with CRE_3 and CRE_4.

## DISCUSSION

We describe five novel *RAX2* pathogenic variants, including both sequence and structural variants, in five apparently unrelated families segregating nonsyndromic recessive or sporadic RP. *RAX2* had previously only been tentatively implicated in autosomal dominant CRD and possibly in AMD.^11,12^ Our findings therefore firmly support the disease-causing role of *RAX2* in IRD, broadening the range of phenotypes and inheritance patterns associated with *RAX2* pathogenic variants, and providing new insight into IRD pathogenetic mechanisms.

To date, pathogenic variants in 61 genes, the majority of which are coding, explain about 60% of ARRP.^28^ The remaining cases are assumed to harbor noncoding sequence or structural variants affecting known IRD genes, missed by exomic approaches, or to carry pathogenic variants in new disease genes. Here, we put forward *RAX2* as a novel disease gene for recessively inherited RP. Previously reported *RAX2*-associated phenotypes were characterized by progressive cone–rod type degeneration, while RP is a rod–cone type of IRD. This may be explained by the fact that different *RAX2* pathogenic variants can differentially alter the transactivation activity of the photoreceptor-specific TFs CRX and NRL. CRX is a homeobox TF essential for the differentiation of both cones and rods, and for the maintenance of normal photoreceptor function through expression modulation of photoreceptor-specific genes.^26^ NRL is a TF that is specifically required for the differentiation of rod photoreceptor cells through the activation of rod-specific genes.^29^ RAX2 is able to bind both to specific TF binding sites and to CRX, which in turn is associated with NRL, forming a heterotrimeric TF complex. The latter activates retinal transcription by recruiting other coactivators and components of the basal transcription machinery.^11^ In addition, RAX2, after CRX and NRL, is one of the top expressed TFs in the adult human retina, which suggests a major role in the regulation of retinal transcription.^30^ Given the expression and function of NRL in rods, we hypothesize that the *RAX2* variants identified here predominantly affect the transactivating activity of NRL thus resulting in a rod–cone instead of a cone–rod phenotype.

The four previously reported *RAX2* variants were identified in patients with CRD or AMD. Segregation was demonstrated for one variant, c.465_475del, consistent with autosomal dominant inheritance.^12^ The heterozygous c.417_422dup variant identified in a CRD patient has a minor allele frequency (MAF) of 0.08% in gnomAD and was also present in the proband’s mother and two siblings said to have normal vision, challenging the pathogenicity of this heterozygous variant. For two variants, c.409G>C and c.260G>A, respectively identified in a single CRD and AMD patient, no segregation analysis was performed.^11^ These four variants were reported to cause IRD as dominant alleles that affect photoreceptor gene transactivation, resulting in a potential dominant-negative effect. Indeed, two variants were demonstrated to exhibit increased transactivation activity.^11^ Here, biallelic *RAX2* variants underlie an RP phenotype, expected to lead to loss of function, due to the absence of protein synthesis or the disruption of functional domains. This is supported by the protein structure modeling of RAX2, where the recessive p.(Ser49Pro) and p.(Pro52Arg) variants, both located in the highly conserved homeodomain, are shown to greatly disrupt RAX2 folding and/or stability. In contrast, modeling RAX2 as a RAX2/CRX heterodimer bound to DNA demonstrates the effect of the dominant p.(Arg87Gln) variant on homeodomain/DNA higher-order complex formation, while no effect on protein stability or DNA-binding is expected. Moreover, no cases of homozygous loss of function variants in *RAX2* are present in the gnomAD database. Interestingly, the occurrence of both dominant and recessive IRD phenotypes caused by pathogenic variants in the same gene has been reported for other retinal TFs. Pathogenic alleles in *CRX* are mainly associated with dominant CRD, RP, and Leber congenital amaurosis (LCA),^31,32^ but rare biallelic *CRX* variants have also been found in LCA.^32^ Both dominant and recessive *NRL*-associated RP could be explained by gain of function and loss of function of *NRL*, respectively.^33^ Finally, a specific missense variant in *NR2E3* was found to lead to autosomal dominant RP by a dominant-negative effect, while all other *NR2E3* variants reported have been associated with autosomal recessive IRD and enhanced S-cone syndrome.^34^

Apart from the novel missense variants in *RAX2*, both a partial and complete gene deletion of *RAX2* (chr19:3,771,337-3,774,298 and chr19:3,765,788-3,772,920) were identified, suggesting a predisposition of this region to structural variation. The partial deletion spans the start codon of the gene and is therefore expected to result in complete loss of function. The current findings highlight the importance of CNV analysis, which was needed to solve two of the five families, for an accurate genetic diagnosis in IRD patients. In addition, one *RAX2* frameshift variant, c.335dup p.(Ala113Glyfs*178), was identified in two patients of Belgian origin. Haplotype analysis suggested a common ancestry of this variant. This variant is assumed to escape NMD and therefore may give rise to a truncated read-through RAX2 protein with a disrupted C-terminal region. A dominant-negative effect is not expected for c.335dup however, because the parents of both patients I and II do not display a retinal phenotype while being heterozygous for the variant. RAX2 orthologues contain a C-terminal OAR domain, the function of which is presumed to be important for transactivation, protein–protein interactions, or DNA-binding. Although this domain seems to be weakly conserved in human RAX2, other highly conserved amino acids are present in the C-terminal region downstream of c.335dup (Fig. 3a). It has indeed been proposed that other segments within the C-terminal region of RAX2 orthologues but outside the OAR domain are involved in transactivation.^8^

In an earlier study, in vitro luciferase assays demonstrated that the transactivation activity of RAX2 was able to reach a peak depending on the amount of *RAX2* plasmids used.^11^ In addition, a more recent study reported interspecies variation in the expression of the *RAX* orthologue in African cichlid fishes, resulting in variable opsin expression and visual system diversity.^35^ This suggests the need for an optimal protein level for members of the Rax family to function in photoreceptor gene transactivation. The function of RAX2 and other retinal TFs therefore appears to be sensitive to alterations in gene dosage levels, leading to the deregulation of photoreceptor gene expression, with subsequent photoreceptor degeneration.

The observation that pathogenic variants in the same transcriptional coactivator gene can result in distinct retinal phenotypes with different inheritance patterns also reflects the growing consensus that regulatory networks of retinal gene expression, defined by precise temporal and spatial patterns, are so complex that closely located perturbations can result in different phenotypic manifestations. Further disease modeling and functional studies will provide more mechanistic insight into the role of *RAX2* loss of function in autosomal recessive IRD. However, neither rodents nor lagomorphs harbor a *RAX2* orthologue. *Xenopus tropicalis* on the other hand has a *RAX2* orthologue, *rx-l*, and has an eye with the same major cell types as the human eye. This therefore seems an excellent model organism in which to further study *RAX2* loss of function and its associated phenotypes.^36^ Interestingly, morpholino-based knockdown of *rx-l* in *Xenopus* appeared to impair late retinogenesis and reduce photoreceptor-specific gene expression.^8^ This is in line with the phenotype observed in human IRD due to biallelic *RAX2* variants.

To conclude, we found biallelic pathogenic variants in *RAX2* to be associated with ARRP, revealing *RAX2* as a novel gene for recessively inherited rod-dominated retinal diseases. The identification of *RAX2* biallelic pathogenic variants in five families of European origin indicates that this gene may underlie a nonnegligible fraction of ARRP cases of other populations that still lack a molecular diagnosis. The *RAX2* mutational spectrum was broadened from sequence to structural variants. Moreover, the identification of pathogenic structural variants in *RAX2* stresses the importance of CNV assessment in exome and genome sequencing data in IRD.

## Electronic supplementary material

Supplementary data
