# Supplementary material for: Biallelic sequence and structural variants in RAX2 are a novel cause for autosomal recessive inherited retinal disease
Source: Genet Med. 2018 Oct 31;21(6):1319–29. doi: 10.1038/s41436-018-0345-5 (PMC6752271; doi:10.1038/s41436-018-0345-5)
Supplement: Supplementary file 1 — Supplementary data [file 41436_2018_345_MOESM1_ESM.docx]

**SUPPLEMENTARY DATA**

**Suppl. Table 1 - Primers used for *RAX2* variant confirmation, segregation analysis, and deletion breakpoint analysis.**

| **Patient I, II (c.335dup)** | | | |
| --- | --- | --- | --- |
| RAX2_c.355dup_F | ACCACGGTTGCTCCAT | RAX2_c.355dup_R | GGAACGCTTGGGAGATC |
| **Patient III (g.3771337_3774298del)** | | | |
| RAX2_del1_F | TGTTACCCACACCATTCTCTGC | RAX2_del1_R | CCCTCTCCTTTCCATCTCTAG |
| **Patient IV (c.145T>C)** | | | |
| RAX2_c.145C>T_F | CCCCAGTGGAGGGAAC | RAX2_c.145C>T_R | GTCAAAACCTCAGCTCCCAC |
| **Patient V, VI (c.155C>G)** | | | |
| RAX2_c.155C>G_F | CAGCCTGGGGATGCAGAG | RAX2_c.155C>G_R | TCCTAGCTTCTCTTCTGCTGT |
| **Patient V, VI (g.3765788_3772920del)** | | | |
| RAX2_del2_F | AAGGAAGGGCAGGATGTACC | RAX2_del2_R | CACGAGCCTAGGGAGAAA |
| RAX2_del2_seq | AGAGGGCTGTGAGGAATTCA |  |  |

**Suppl. Table 2 - Primers used for *RAX2* c.335dup haplotype analysis.**

| RAX2_SNP1_F | tatctgcgggtttcttgtcc | RAX2_SNP1_R | aaactttgcattatttttcttcttg |
| --- | --- | --- | --- |
| RAX2_SNP2_F | GCCCCTCAGTAGGTGATCTG | RAX2_SNP2_R | gtggagaccaggggtgtgt |
| RAX2_SNP3_F | aagccaggcaacgtgactac | RAX2_SNP3_R | ggggtgtgaggtctcagg |
| RAX2_SNP4_F | ccactaagctcagctaattaaaaagg | RAX2_SNP4_R | gacagggctgtgctgaaata |
| RAX2_SNP5_F | GCTGCCCCACTAACCATCT | RAX2_SNP5_R | TGCAATATCTGTCTGTCTGTCTG |
| RAX2_SNP6_F | agctcgggctcagacctc | RAX2_SNP6_R | CGTCCCGCTCTCTTACGG |
| RAX2_SNP7_F | tggggacatcagactgtgag | RAX2_SNP7_R | agggtgggactgagtcagatt |
| RAX2_SNP8_F | GGGATGGGCACTTGGATG | RAX2_SNP8_R | ACTCTGGACAGCTCCACGAT |
| RAX2_SNP9_F | gaattccacaaccctgaacc | RAX2_SNP9_R | actccccaccctggcttc |
| RAX2_SNP10_F | ccacaactagggttcccaga | RAX2_SNP10_R | ctccctggtctcaggttgtc |
| RAX2_SNP11_F | TGTCTGTTACAACCTCCGTATGA | RAX2_SNP11_R | GTGGGCTTGTCCTGATGG |
| RAX2_SNP12_F | ctcactttggggctcgaata | RAX2_SNP12_R | cgagggggtgactgttttta |
| RAX2_SNP13_F | cagagccttttgtggacctc | RAX2_SNP13_R | cctcagtctccccgttagaa |
| RAX2_SNP14_F | ggagcccctgtgtctattagc | RAX2_SNP14_R | acaaacccacaggctcagac |
| RAX2_SNP15_F | ccctgcacacctacCTGTTT | RAX2_SNP15_R | gcagggaacatctcactggt |
| RAX2_SNP16_F | aaggaaggctcctcaacagg | RAX2_SNP16_R | tcagggtctttcgagtggag |
| RAX2_SNP17_F | ccgtattcattatataactgggaaaaa | RAX2_SNP17_R | cgccttaggaatttattctgg |
| RAX2_SNP18_F | agctgagtttccatccttgg | RAX2_SNP18_R | ggggtcaggcttcctctc |
| RAX2_SNP19_F | aacagggacagggtgcag | RAX2_SNP19_R | CTCGAAGATCCTGAGCGAGT |
| RAX2_SNP20_F | ggtagagactgggccctgag | RAX2_SNP20_R | ggtagctatgctgggactgg |
| RAX2_SNP21_F | aaggtgcacttttccttttct | RAX2_SNP21_R | catgtatttaccacaattttaaaaaga |
| RAX2_SNP22_F | ctgctgcgaaactggagaag | RAX2_SNP22_R | gggatgtgggtgtgttttg |
| RAX2_SNP23_F | tgcagggtcaaatgtctgtt | RAX2_SNP23_R | gccttgaggtgttttgaacc |
| RAX2_SNP24_F | tggcttcagatggacacaaa | RAX2_SNP24_R | aagggcttccaggtcagtg |
| RAX2_MS1_F | cagagagtgggtccccaaa | RAX2_MS1_R | ggcctcttattccctggtg |
| RAX2_MS2_F | agtgggagagactgagacatca | RAX2_MS2_R | actcagtttcccgaatcacg |
| RAX2_MS3_F | tgttcacctactacctacgtaacaaaa | RAX2_MS3_R | gaccaaagtctggcagggta |

**Suppl. Table 3 - Overview of the number of cases from the European IRD cohort that underwent exome sequencing.**

| **Contributing team** | **N° of cases studied** |
| --- | --- |
| Belgium (Ghent) | 300 |
| UK (Leeds) | 277 |
| Spain (Madrid) | 1350 |
| Italy (Naples) | 159 |
| **TOTAL** | **2086** |

**Suppl. Table 4 - Overview of (likely) pathogenic biallelic variants in patients with *RAX2*-associated ARRP.** Variants are annotated by Alamut Batch. CADD Phred scores are provided in the last column. An overview of the exome sequencing filtering strategies is given in **Suppl. File 3**.

| **patient** | **zygosity** | **gene** | **codingEffect** | **gNomen (GRCh37)** | **cNomen** | **pNomen** | **gnomAD** | **CADD** |
| --- | --- | --- | --- | --- | --- | --- | --- | --- |
| patient I | het | *FAM198B* | missense | chr4:g.159052122G>A | NM_001031700.2:c.1192C>T | p.Arg398Cys | 0.000112 | 33.0 |
|  | het | *FAM198B* | missense | chr4:g.159076894A>G | NM_001031700.2:c.1018T>C | p.Trp340Arg | 0.000016 | 32.0 |
|  | het | *LILRB2* | stop gain | chr19:g.54782901del | NM_005874.4:c.721del | p.Leu241* | / | 22.2 |
|  | het | *LILRB2* | frameshift | chr19:g.54782905insC | NM_005874.4:c.716insG | p.Ser240Lysfs*9 | / | 17.3 |
|  | **hom** | ***RAX2*** | **frameshift** | **chr19:g.3770839dup** | **NM_032753.3:c.335dup** | **p.Ala113Glyfs*178** | **0.000008** | **25.0** |
| patient II | het | *FSIP2* | missense | chr2:g.186627964C>A | NM_173651.3:c.1295C>A | p.Thr432Lys | / | 21.0 |
|  | het | *FSIP2* | missense | chr2:g.186667440A>T | NM_173651.3:c.13407A>T | p.Leu4469Phe | 0.000212 | 24.7 |
|  | het | *KIAA0368* | missense | chr9:g.114134037G>A | NM_001080398.1:c.5135C>T | p.Ser1712Phe | 0.000047 | 32.0 |
|  | het | *KIAA0368* | missense | chr9:g.114134852C>T | NM_001080398.1:c.4919G>A | p.Arg1640Gln | 0.000024 | 23.6 |
|  | hom | *MAGIX* | frameshift | chrX:g.49021346del | NM_024859.3:c.425del | p.Gly142Alafs*4 | 0.000017 | 24.1 |
|  | het | *NFX1* | missense | chr9:g.33318937C>T | NM_001318758.1:c.1718C>T | p.Ser573Leu | 0.000227 | 23.4 |
|  | het | *NFX1* | missense | chr9:g.33351561C>T | NM_001318758.1:c.2431C>T | p.Arg811Trp | 0.000008 | 32.0 |
|  | **hom** | ***RAX2*** | **frameshift** | **chr19:g.3770839dup** | **NM_032753.3:c.335dup** | **p.Ala113Glyfs*178** | **0.000008** | **25.0** |
| patient III | hom | *BAG3* | missense | chr10:g.121432086G>A | NM_004281.3:c.827G>A | p.Arg276Gln | 0.000094 | 23.6 |
|  | hom | *CASP7* | missense | chr10:g.115457334C>T | NM_001267056.1 :c.82C>T | p.Arg28Trp | 0.000105 | 24.4 |
|  | hom | *DHRS7B* | missense | chr17:g.21075488G>A | NM_015510.4:c.178G>A | p.Ala60Thr | 0.000049 | 27.6 |
|  | hom | *GPR26* | missense | chr10:g.125434339G>A | NM_153442.3:c.674G>A | p.Arg225Gln | 0.000020 | 24.6 |
|  | het | *LTBP2* | missense | chr14:g.74976028C>T | NM_000428.2:c.3316G>A | p.Gly1106Arg | 0.000033 | 23.0 |
|  | het | *LTBP2* | missense | chr14:g.75078503C>T | NM_000428.2:c.145G>A | p.Asp49Asn | / | 22.6 |
| patient IV | **hom** | ***RAX2*** | **missense** | **chr19:g.3771596A>G** | **NM_032753.3:c.145T>C** | **p.Ser49Pro** | **/** | **25.4** |
| patient V | het | EYS | missense | chr6:g.64940625G>A | NM_001292009.1:c.6284C>T | p.Pro2095Leu | 0.000464 | 15.4 |
|  | het | *EYS* | missense | chr6:g.65300660T>A | NM_001292009.1:c.5100A>T | p.Lys1700Asn | 0.000020 | 24.7 |
|  | het | *JAK2* | splice site | chr9:g.5073696A>T | NM_001322194.1:c.1777-2A>T | p.? | 0.000026 | 24.6 |
|  | het | *JAK2* | splice site | chr9:g.5073697G>T | NM_001322194.1:c.1777-1G>T | p.? | 0.000019 | 25.7 |
|  | het | *JAK2* | missense | chr9:g.5073699C>T | NM_001322194.1:c.1778C>T | p.Ser593Phe | 0.000007 | 33.0 |
|  | het | *PRUNE2* | missense | chr9:g.79244204G>A | NM_015225.2:c.9053C>T | p.Ser3018Leu | 0.000034 | 34.0 |
|  | het | *PRUNE2* | splice site | chr9:g.79244208T>A | NM_015225.2:c.9051-2A>T | p.? | 0.000452 | 35.6 |
|  | **hom** | ***RAX2*** | **missense** | **chr19:g.3771586G>C** | **NM_032753.3:c.155C>G** | **p.Pro52Arg** | **/** | **26.9** |
|  | het | *TTN* | missense | chr2:g.179408636C>T | NM_001267550.2:c.96235G>A | p.Asp32079Asn | 0.000181 | 23.9 |
|  | het | *TTN* | missense | chr2:g.179580288C>T | NM_001267550.2:c.25853G>A | p.Gly8618Glu | 0.000049 | 23.0 |
|  | het | *TTN* | missense | chr2:g.179606622C>T | NM_001267550.2:c.11338G>A | p.Glu3780Lys | 0.000137 | 17.6 |

**Suppl. Table 5 - Summary of the bioinformatics analyses of the identified *RAX2* deletion breakpoint regions.**

|  | **Patient III** | **Patient V, VI** |
| --- | --- | --- |
| Chr | 19 | 19 |
| Start (hg19) | 3,771,337 | 3,765,788 |
| End (hg19) | 3,774,298 | 3,772,920 |
| Microhomology (bp) | 3 | 4 |
| **5' breakpoint region** | | |
| Repetitive element | L2c | *Alu*Sx |
| Number of sequence motifs | 15 | 11 |
| **3' breakpoint region** | | |
| Repetitive element | - | *Alu*Sx1 |
| Number of sequence motifs | 17 | 12 |
| **Conclusion** | | |
| Potential molecular mechanism | Replicative/NHEJ | Replicative/NHEJ/NAHR |

**Abbreviations**: chr, chromosome; E, exon; bp, base pair; NA, not applicable; NHEJ, non-homologous end-joining; NAHR, non-allelic homologous recombination. Replicative stands for replicative-based mechanisms and includes fork stalling and template switching (FoSTeS), microhomology-mediated break-induced replication (MMBIR) and serial replication slippage (SRS).

**Suppl. File 1 - Details of ethical statements.**

Ethical approval was given by the local ethics committee of Ghent University Hospital (Belgium; B670201734438), Università degli Studi della Campania “Luigi Vanvitelli” (Italy; 0008189/2015), Fundación Jimenez Díaz (Spain; PIC 134-2016_FJD), and Leeds East Research Ethics Committee (UK; 17/YH/0032 and IRAS project ID 167881).

**Suppl. File 2 - Detailed description of fundus and OCT imaging in patients with *RAX2*-associated ARRP.**

**Patient I**

Pale optic disc, very narrow to non-existent arterial vessels, central foveal island of spared retina with yellow discoloration, a peripapillary ring of atrophy and pigment, and atrophy of the (mid-)periphery with both spicular and nummular intraretinal pigment.

Central foveal island with sparing of photoreceptor layers and loss of outer retinal layers outside the (para-)foveal area.

**Patient II**

Pale optic disc, narrow retinal vasculature, and patchy grey areas of atrophic retina in a circular band outside the vascular arcades in the mid-periphery. Very limited intraretinal pigment migration in the peripheral retina and cellophane maculopathy.

Central foveal island with sparing of photoreceptor layers and loss of the outer retinal layers outside the (para-)foveal area.

**Patient III**

Rose-colored optic disc, narrow retinal vessels and an atrophic retinal pigment epithelium in the mid-periphery with rare bone spicule pigmentation.

Preserved retinal layers in the fovea with only small intraretinal cystic changes in the perifoveal region.

**Patient IV**

Pale optic disc, narrow vessels, and chorioretinal atrophy with bone spicule pigmentation, mainly in the inferior periphery, while the macula has a normal appearance.

Preserved outer retinal layers at the great majority of the macular area. A disruption of the ellipsoid band is shown 2000 µm nasally from the foveola. No cystoid macular edema is present. Normal inner retina and choroidal anatomy are shown.

**Patient V, VI**

Classical RP manifestations with widespread spicule type pigmentary changes in the mid-periphery and far peripheral retina. These changes spare the posterior pole and are more extensive in patient V.

Well preserved outer retinal structures in the central macula, with loss of outer nuclear layer volume and ellipsoid zone integrity in the periphery and adjacent to the optic nerve.

**Suppl. File 3 - Overview of exome sequencing filtering strategies.**

The *RAX2*-positive families included in this study were identified by four groups of the European Retinal Disease Consortium (ERDC; https://www.erdc.info). The families were therefore analyzed according to an in-house established exome sequencing filtering strategy, specific for each group. **Patient I, II:** Variants in genes known to cause retinal disease (RetNet panel, https://sph.uth.edu/retnet) were filtered for a minor allele frequency (MAF) < 0.05% (gnomAD, 1000 Genome Project, and an in‑house sequence variation database). The remaining truncating variants (nonsense, frameshift, splice site) and missense variants predicted to be pathogenic (SIFT < 0.05, PolyPhen-2 > 0.8, MAPP < 0.5, Align GVGD > C25, Grantham > 60) were retained and further characterized in Alamut Visual. In parallel, all genes with potentially biallelic variants with a MAF < 0.1% (gnomAD) and a Combined Annotation Dependent Depletion (CADD, v1.3) score ≥ 15 were prioritized for investigation. **Patient III:** All variants with a MAF < 0.1% (gnomAD, ExAC, 1000 Genome Project, and an in‑house sequence variation database) were retained for further analysis. Large homozygous deletions were searched for by identifying regions with a minimal length of 100 bp displaying read coverage < 5. **Patient IV:** Variants in a virtual panel of 194 IRD-associated genes with a MAF < 5% (gnomAD) were retained. Missense variants were retained if they met the following *in silico* prediction tool criteria: SIFT < 0.05, PolyPhen-2 > 0.8, M-CAP > 0.025, CADD > 10, Align GVGD > C15. Conservation of the protein residue was also assessed by GERP > 2, PhyloP > 0.8, Grantham and PhastCon > 0.8. **Patient VI, VI:** Variants present in gnomAD with a MAF ≥ 0.1% were excluded using vcfhacks perl scripts written by Dr David Parry (<https://github.com/gantzgraf/vcfhacks>). Variants present in ≥ 3 unrelated individuals in the same sequencing lane (eleven individuals total) were excluded to remove sequencing artefacts. The remaining variants were annotated using NCBI's Variant Effect Predictor. Genes with potentially biallelic variants with a CADD score of ≥ 15 were prioritized for investigation. Of these, variants in known IRD genes, identified using RetNet (https://sph.uth.edu/retnet/) were highlighted for segregation analysis. Copy number variants were identified using the R package ExomeDepth (Plagnol et al., 2012). Ten bam files from unrelated samples prepared in the same batch and sequenced in the same lane were used as controls. Calls not recorded as “normal variation” in Conrad et al. (2010) were prioritized for inspection and were ranked by Bayes factor.

**Suppl. Figure 1 - Fundus autofluorescence images of patients with *RAX2*-associated ARRP (left eye).**

**Patient I**

**
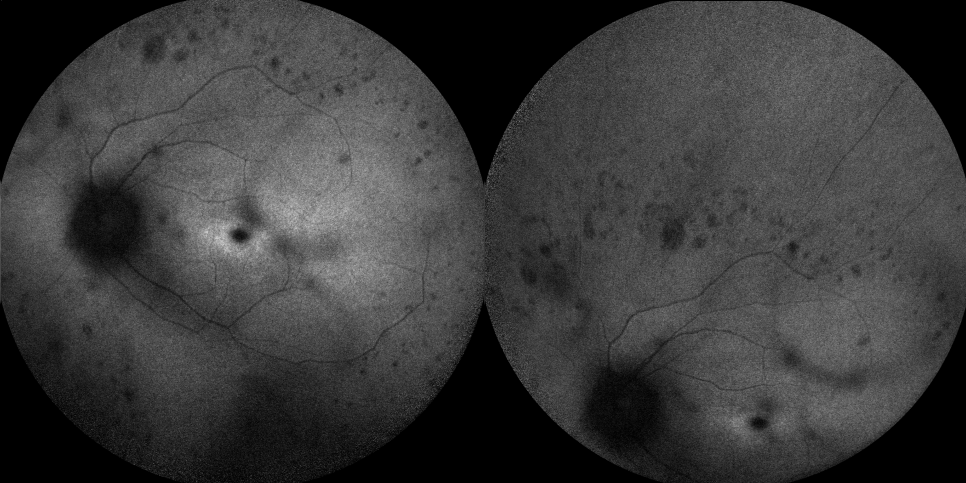
**

**Patient II**

**
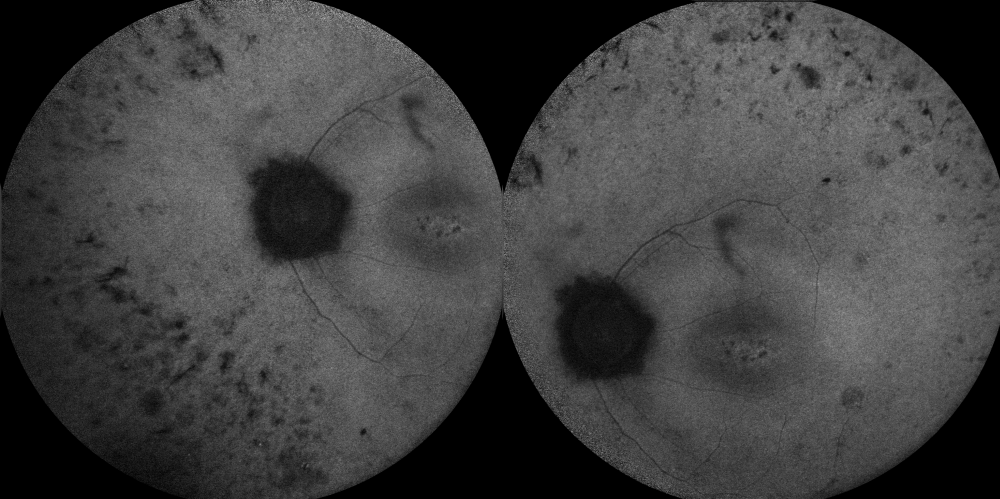
**

**Patient III**

**
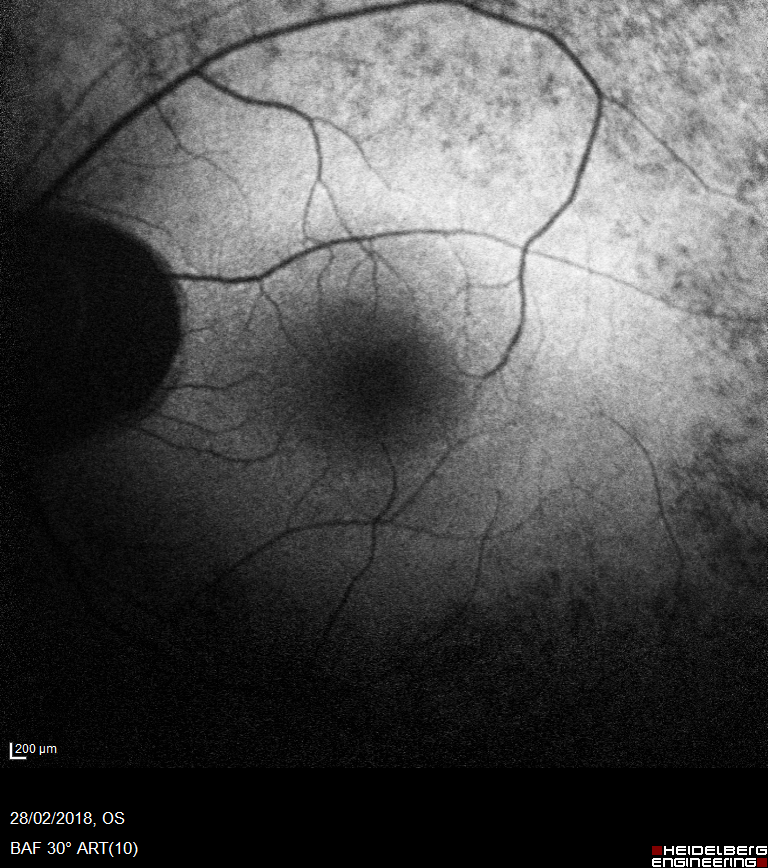
**

**Patient IV**

**
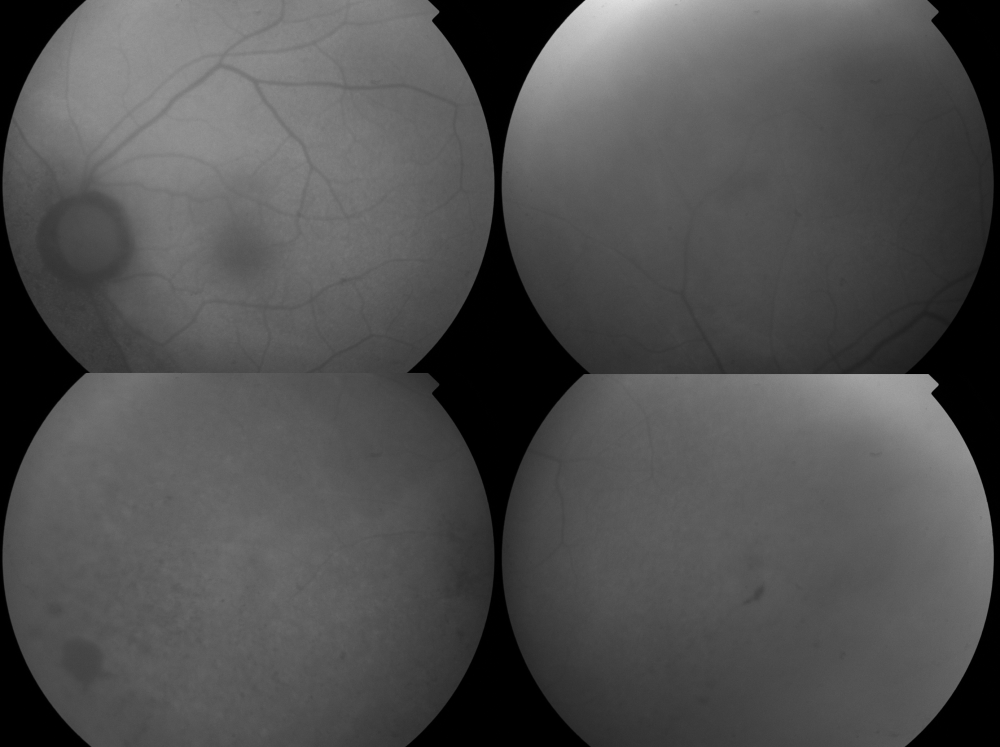
**

**Patient V**

**
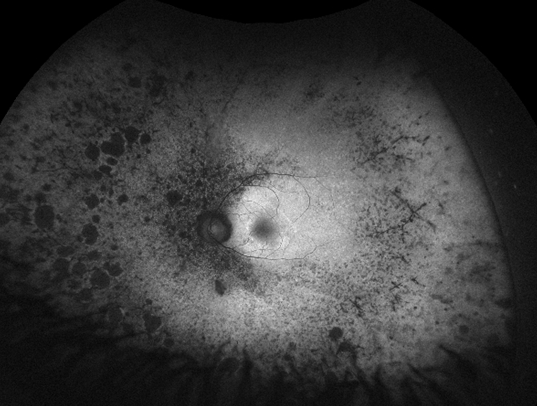
**

**Patient VI**

**
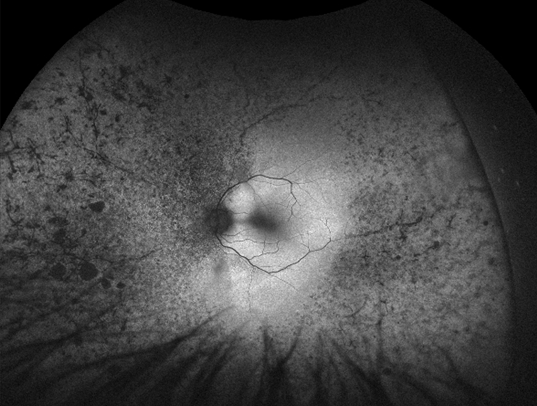
**

**Suppl. Figure 2 - ERG waveforms recorded from three patients (II, III, and IV) with *RAX2*-associated ARRP (left eye).**

**Patient II**


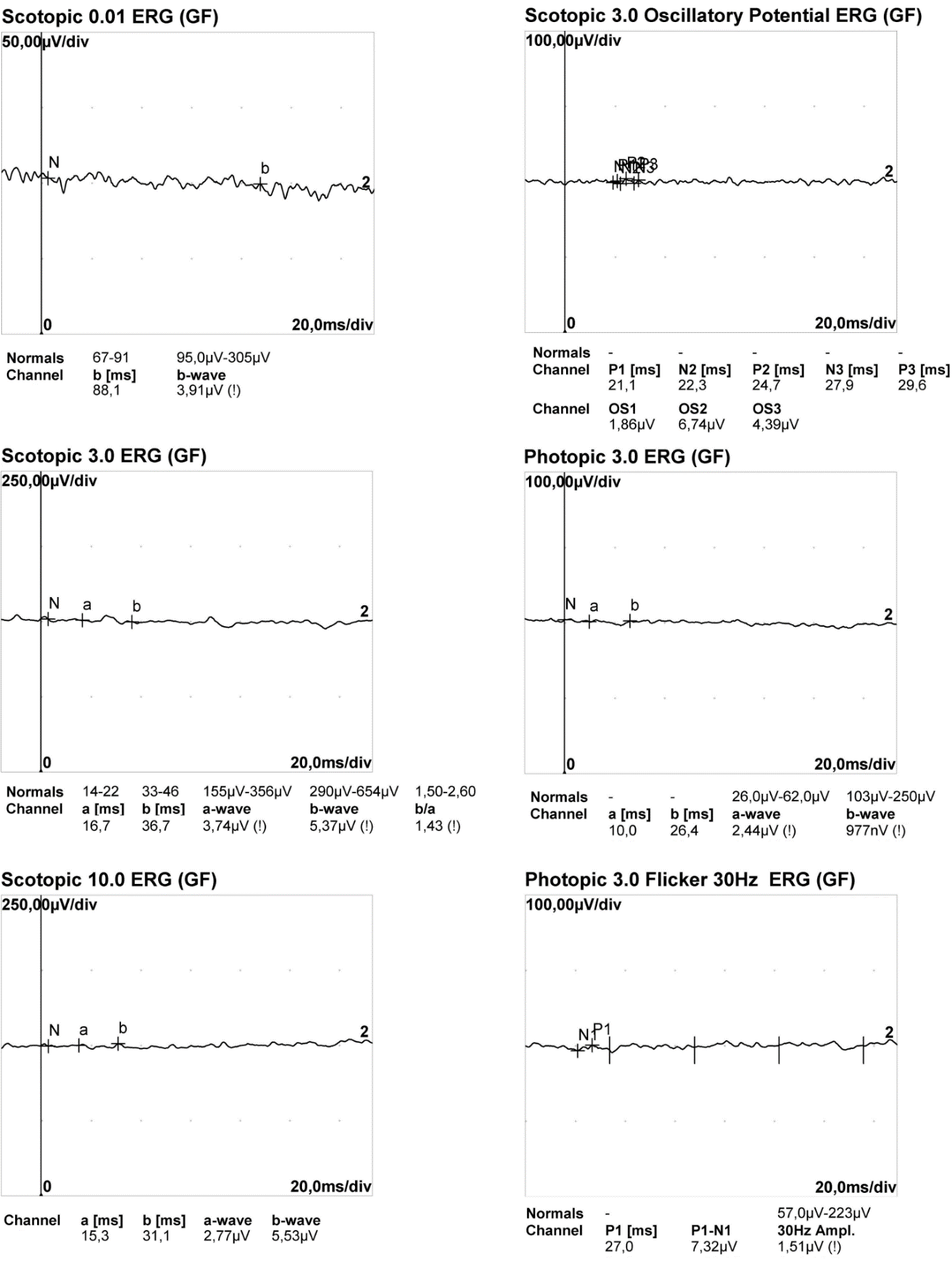


**Patient III**


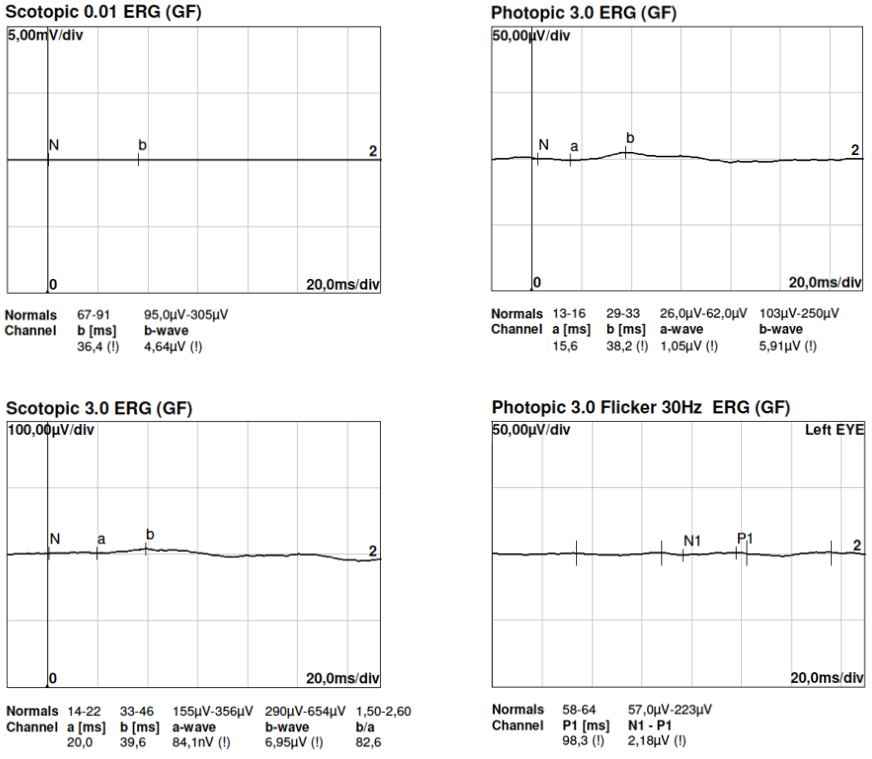


**Patient IV**


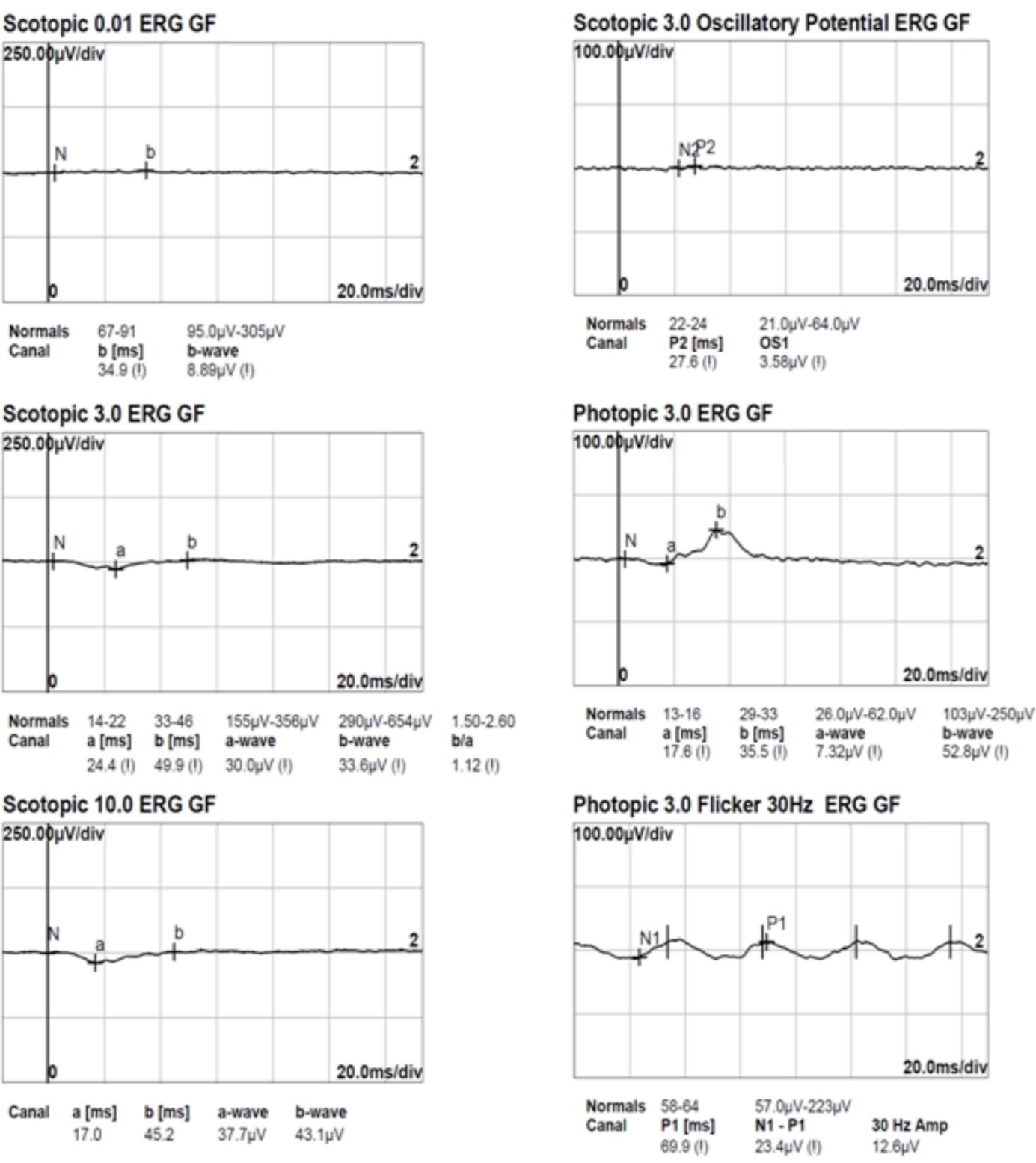


**Suppl. Figure 3 - Results of ophthalmic evaluation of family members of patients II and III, being carriers of heterozygous *RAX2* variants. (A)** The mother (II:5) of patient II, who is heterozygous carrier of the c.335dup *RAX2* variant, was examined at the age of 55. Left (OS) and right (OD) blue laser autofluorescence (BAF) and infrared reflectance (IR) images display a normal fundus with a normal optic disc, regular retinal vasculature, and the absence of intraretinal pigment in the periphery.

The brother (II:3) and sister (II:4) of patient III, who were found to be heterozygous for the partial *RAX2* deletion, underwent full ophthalmological evaluation also including fundus autofluorescence (B), OCT (C), and standard ERG) (D) at the age of 52 and 49 respectively. Since this assessment did not reveal any sign of retinal disease, no further tests were carried out.

**A. Ophthalmological assessment of the mother (II:5) of patient II.**

**
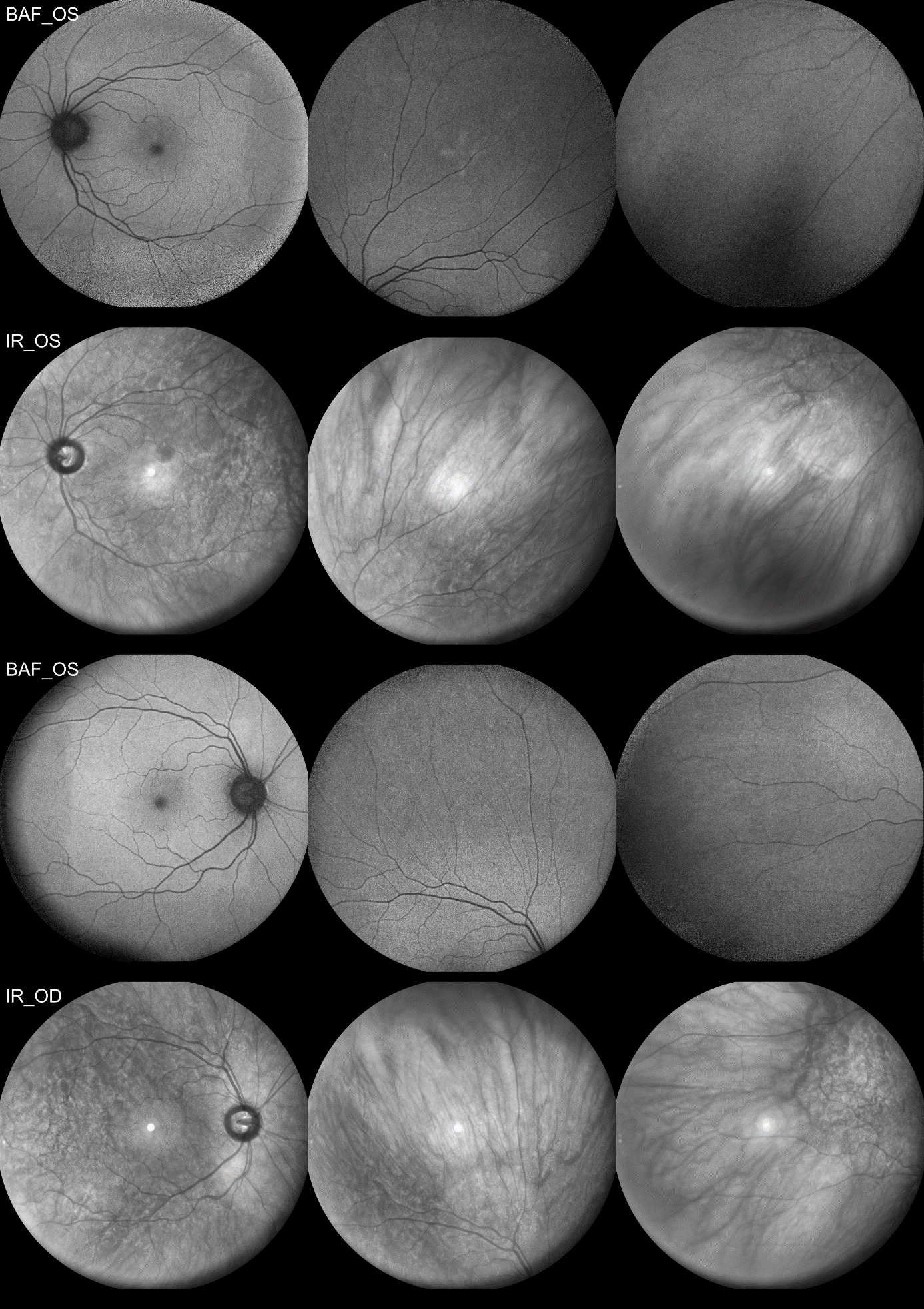
**

**B. Fundus autofluorescence images of right and left eye in II:3 and II:4.**


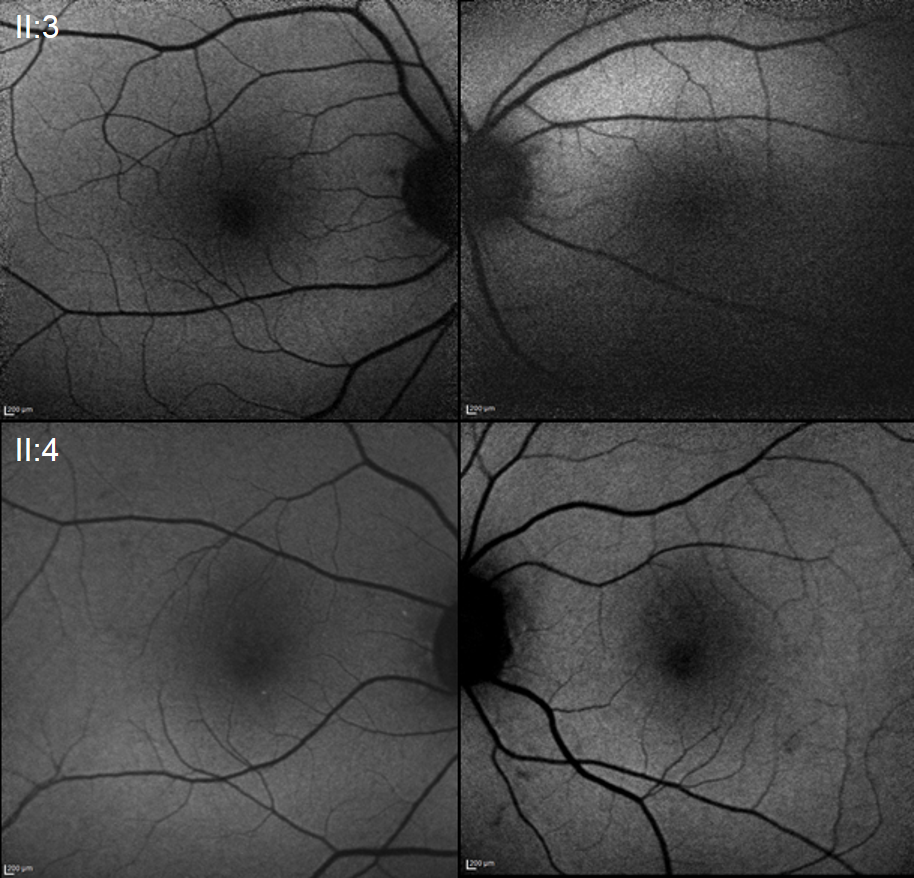


**C. OCT images of right and left eye in II:3 and II:4.**


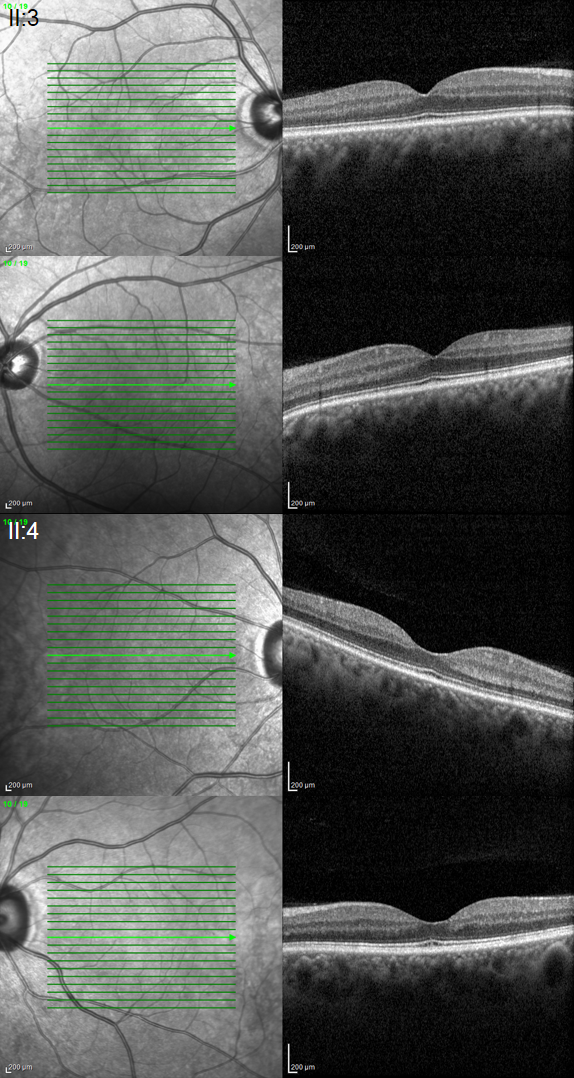


**D. ERG waveforms of right and left eye in II:3 and II:4.**

**II:3**


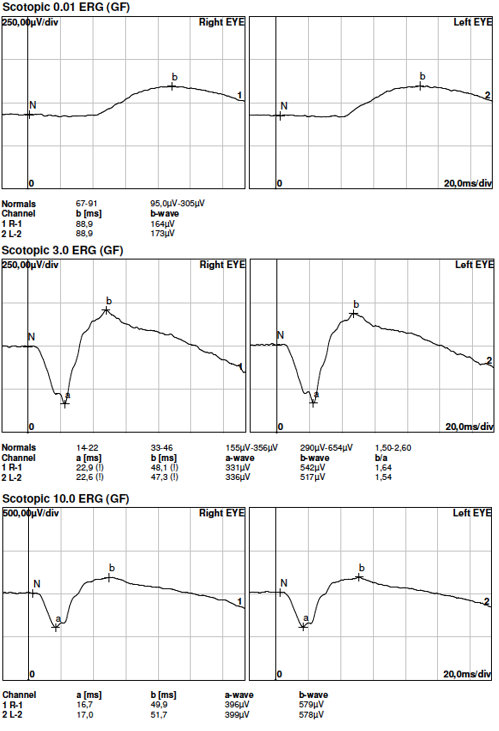


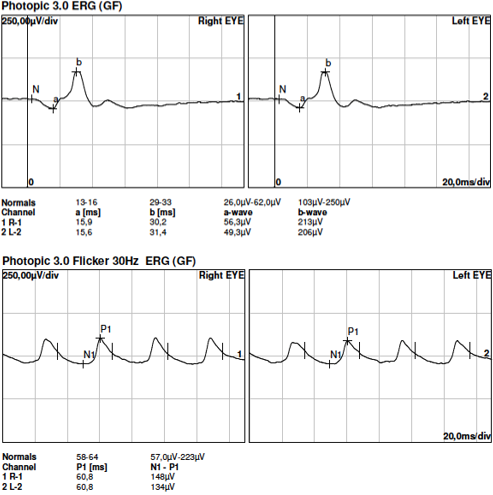


**II:4**


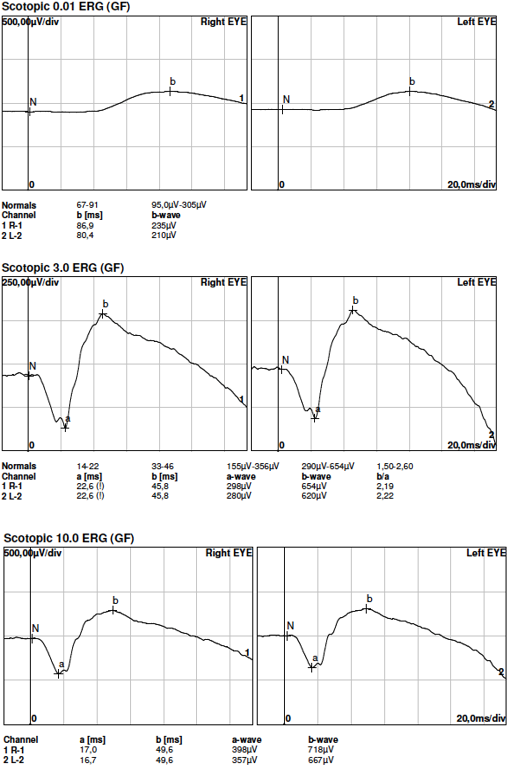


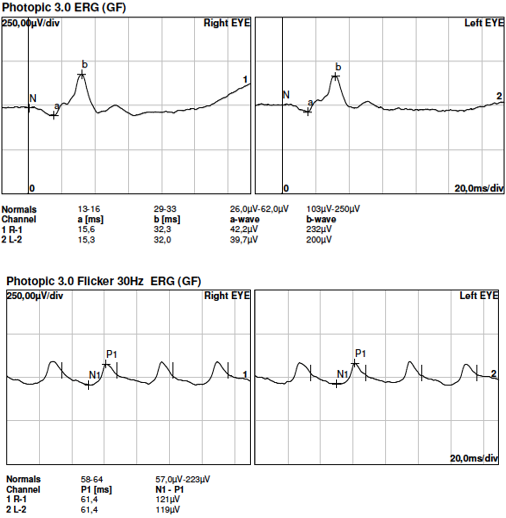


**Suppl. Figure 4 - Results of CNV analysis of the *RAX2* deletion in siblings of patients V and VI using qBase+.** Individuals II:2 and II:3 are the unaffected sisters of patients V and VI respectively. RAX2_CNV1 and RAX2_CNV2 primers are located within the deleted region (g.3765788_3772920del). Primer sequences are given below the figure. Two reference genes *ZNF80* and *GPR15* were used for normalization of the relative quantities and two positives controls C1 and C2 with known copy number were used as a reference to calculate the copy numbers.

**
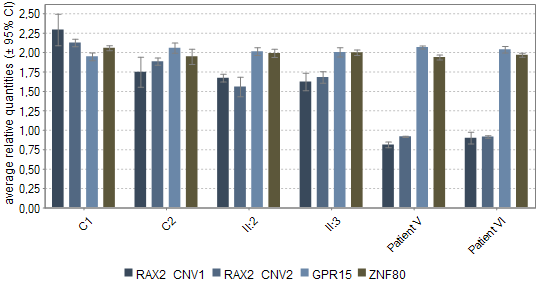
**

| RAX2_CNV1_F | GCCTTTGCTCCCACCTT | RAX2_CNV1_R | AGAGCCTGTGCATGTTCCTT |
| --- | --- | --- | --- |
| RAX2_CNV2_F | GGATCCTGCCTTCCTTGAC | RAX2_CNV2_R | TTTCTCTTCCCCTCTCTGTGA |

**Suppl. Figure 5 - Results of SNP and microsatellite genotyping in two patients and four carriers of the *RAX2* c.335dup variant.**

**
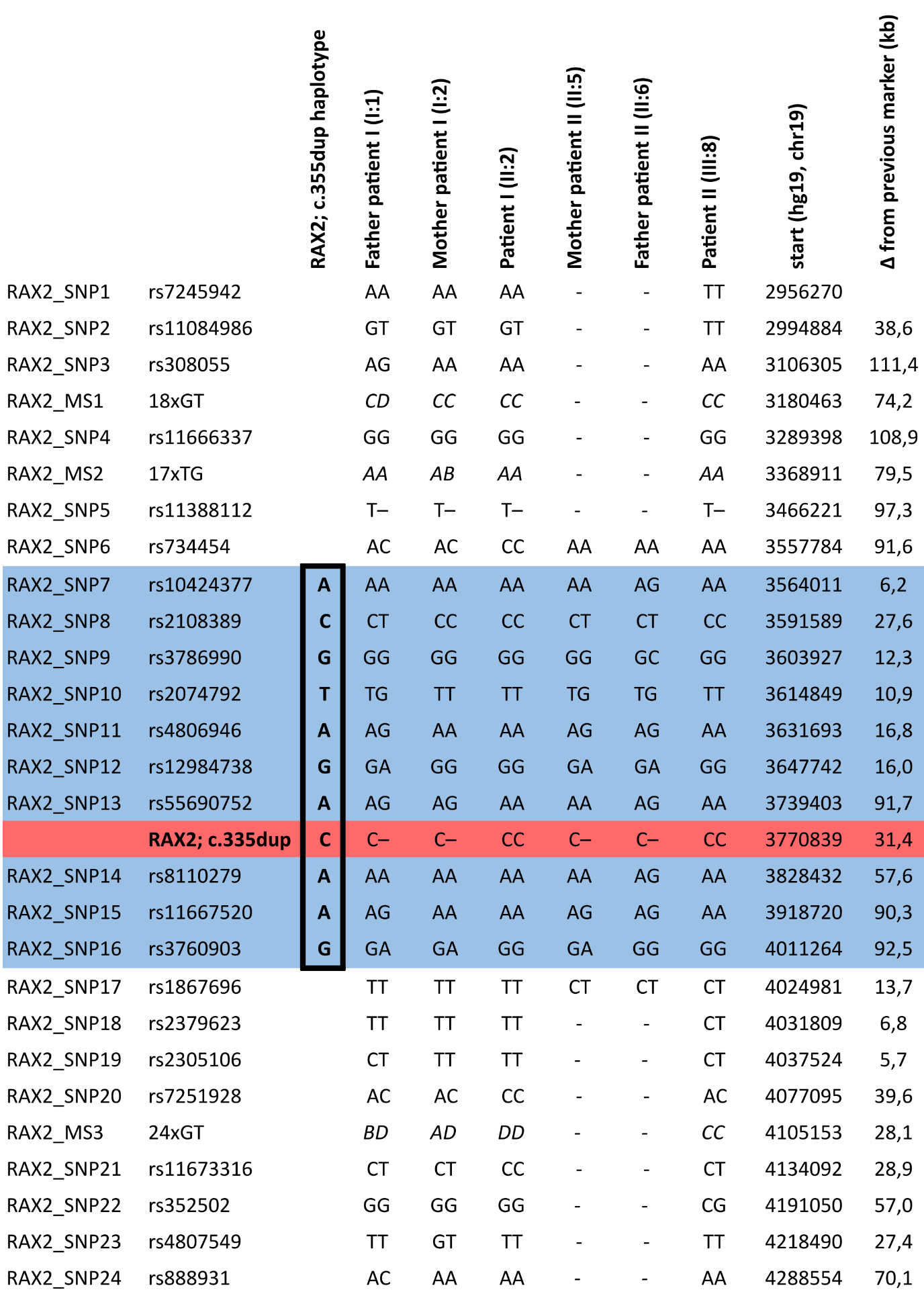
**

**Suppl. Figure 6 - Based on homology models for the RAX2 homeodomain, the p.(Ser49Pro) and p.(Pro52Arg) variants are expected to affect the folding and/or stability of the RAX2 protein. (A)** Ramachandran plot of Ser49 in the RAX2 model. The probability contours for proline are indicated. The phi/psi values of Ser49 are incompatible with a proline. **(B)** The hydrophobic ring of Pro52 inserts into the hydrophobic core of the RAX2 homeodomain. Residues forming hydrophobic cage around this proline are indicated.

**A**

**
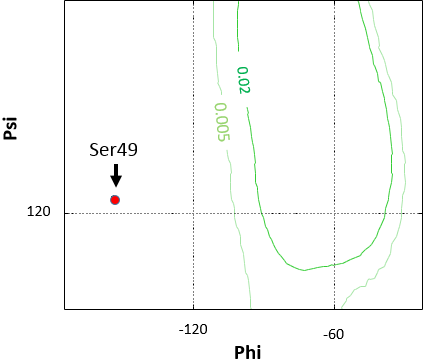
**

**B**

**
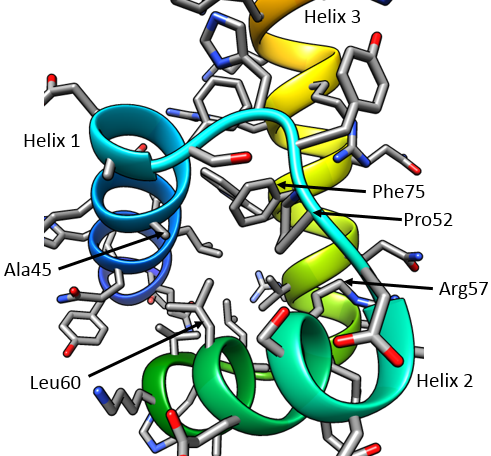
**

**Suppl. Figure 7 - Visualization of the identified *RAX2* deletions with details of the presence of microhomology, repetitive elements and sequence motifs at the breakpoint regions. (A)** Both CNVs are visualized in the UCSC Human Genome Browser demonstrating an overlap with the *RAX2* coding region. **(B)** Multiple sequence alignments. Sequences of 150 bp surrounding the junctions of each deletion were aligned to the proximal and distal reference sequences using ClustalW. The proximal and distal reference sequences are shown in blue and green respectively. The junction sequences are depicted in the color of the reference sequence they align with. Microhomology between the proximal and distal reference sequence and the junction are shown in red (Verdin et al. 2013). **(C)** For the two CNVs that have been delineated at nucleotide level both breakpoint regions are shown in the UCSC genome browser displaying the RepeatMasker track. **(D)** The *Alu*-repeats overlapping the breakpoints of the complete *RAX2* deletion were aligned using BLAST to determine the percentage of sequence identity between them. **(E)** For both CNVs the presence of 40 previously described sequence motifs (Abeysinghe et al. 2003) has been assessed in both breakpoint regions using Fuzznuc (Rice et al. 2000).

**A - Visualization of *RAX2* deletions in the UCSC Human Genome Browser**

**
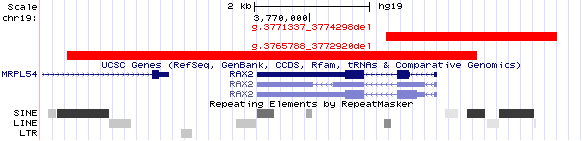
**

**B - Microhomology at CNV breakpoints**

***RAX2* E1-E2 deletion: chr19:3771337-3774298**

Proximal ctccttcacccctggctcaagggaaacgcctccaacttaacccctccatg
Deletion ctccttcacccctggctcaagggaaacgcctccaacttaacccctccatg
Distal ggcccattctgggctgtgaggtgggactgtttcagaggcagaggcctgag

Proximal tctttgcacacactgttcaggatGCCtagaaaaactccccttttccttcc
Deletion tctttgcacacactgttcaggatGCCccagagacgagtgccccccaagtc
Distal aggtggagcgggctccaggggaaGCCccagagacgagtgccccccaagtc

Proximal cagctggcacactcctactcaaatgtcaaaacctcagctcccacaccccc

Deletion caaggagccagggggctcaggcaggggagtgccctagagatggaaaggag
Distal caaggagccagggggctcaggcaggggagtgccctagagatggaaaggag

***RAX2* gene deletion: chr19:3765788-3772920**

Proximal catggtgaaacctcgtctctactaaaaatataaaaattagccgggtgtgg
Deletion catggtgaaacctcgtctctactaaaaatataaaaattagccgggtgtgg
Distal acatggcaaaaccctgtctactaaaaatacaaaaattagccaggcgtggt

Proximal tggcacattcctgtagtcccagtTACTtgggaggctgaggcacgagaatt
Deletion tggcacattcctgtagtcccagtTACTcgggaggctgaggcaggagaatt
Distal ggtgggtgcctataactcccagcTACTcgggaggctgaggcaggagaatt

Proximal gcttgagcctgggaggcagaggttgcagtgagctgagatcacgccattgt

Deletion gcttgaacctggtggggtggagcttgcagtgagccaaggtcatgccattg
Distal gcttgaacctggtggggtggagcttgcagtgagccaaggtcatgccattg

**C - Repetitive elements at CNV breakpoints**

***RAX2* E1-E2 deletion: chr19:3771337-3774298**


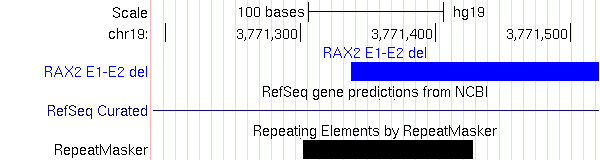


L2c

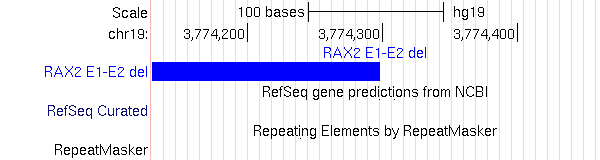


***RAX2* gene deletion: chr19:3765788-3772920**


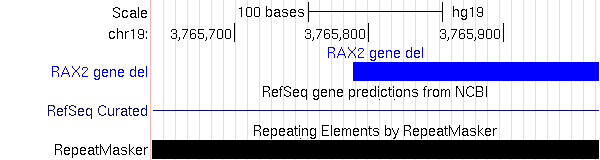


AluSx

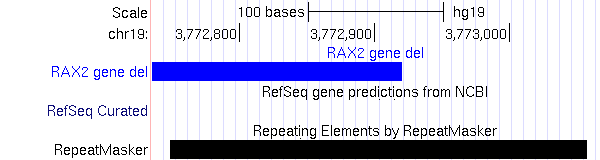


AluSx1

**D - BLAST Alignment of the *Alu*-repeats at the breakpoints of the complete *RAX2* deletion**

| **Score** | **Expect** | **Identities** | **Gaps** | **Strand** |
| --- | --- | --- | --- | --- |
| 313 bits(346) | 4e-90 | 251/298(84%) | 5/298(1%) | Plus/Plus |

AluSx 2 GCTGGGCGCCGTGGCTCACACCTGTAATCCCAGCACTTTGGGAGGC-AAGGCGTTTGGAT 60

|| |||||| ||||||||| ||||||| |||||||||||||||||| ||||| ||||

AluSx1 1 GCCGGGCGCAGTGGCTCACGCCTGTAACCCCAGCACTTTGGGAGGCTGAGGCGGGCGGAT 60

AluSx 61 CACCTGAGGTCAGGAGTTCGAGACCAGCCTGGCCAACATGGTGAAACCTCGTCTCTACTA 120

|||||||| ||| ||||| |||||||||||||||||||||| |||| | | |||||||

AluSx1 61 CACCTGAGATCATGAGTTTGAGACCAGCCTGGCCAACATGGCAAAAC--CCTGTCTACTA 118

AluSx 121 AAAATATAAAAATTAGCCGGGTGTGGTGGCACATTCCTGT-AGTCCCAGTTACTTGGGAG 179

|||||| ||||||||||| || ||||||| | ||| | | |||||| |||| |||||

AluSx1 119 AAAATACAAAAATTAGCCAGGCGTGGTGGTGGGTGCCTATAACTCCCAGCTACTCGGGAG 178

AluSx 180 GCTGAGGCACGAGAATTGCTTGAGCCTGG-GAGGCAGAGGTTGCAGTGAGCTGAGATCAC 238

||||||||| ||||||||||||| ||||| | || ||| ||||||||||| || |||

AluSx1 179 GCTGAGGCAGGAGAATTGCTTGAACCTGGTGGGGTGGAGCTTGCAGTGAGCCAAGGTCAT 238

AluSx 239 GCCATTGTACTCCAGCCTGGGCAACAGAGTGAGACTTGGTCTCAAAAAAAAAAAAAAA 296

||||||| |||||||||||||||||||||||| ||| |||||||||||||||||||

AluSx1 239 GCCATTGCACTCCAGCCTGGGCAACAGAGTGAAACTCCATCTCAAAAAAAAAAAAAAA 296

**E - Sequence motifs at CNV breakpoints**

***RAX2* E1-E2 deletion: chr19:3771337-3774298**

5’ breakpoint region

- Deletion hotspot consensus: 1
- DNA polymerase arrest site: 4
- Ig heavy chain class switch repeat 1: 1
- Ig heavy chain class switch repeat 3: 3
- Ig heavy chain class switch repeat 5: 1
- Murine parvovirus recombination hotspot: 1
- Vaccinia topoisomerase I consensus: 4

3’ breakpoint region

- Deletion hotspot consensus: 3
- DNA polymerase arrest site: 4
- Ig heavy chain class switch repeat 2: 4
- Ig heavy chain class switch repeat 4: 2
- Ig heavy chain class switch repeat 5: 1
- Murine MHC recombination hotspot: 1
- Vaccinia topoisomerase I consensus: 2

***RAX2* gene deletion: chr19:3765788-3772920**

5’ breakpoint region

- ARS consensus *S. cerevisiae*: 1
- Consensus SAR 2: 1
- Consensus SAR 3: 2
- Consensus SAR 4: 2
- Deletion hotspot consensus: 1
- DNA polymerase a/b frameshift hotspot 2: 1
- Ig heavy chain class switch repeat 1: 1
- Ig heavy chain class switch repeat 5: 2

3’ breakpoint region

- Consensus SAR 3: 1
- Deletion hotspot consensus: 2
- DNA polymerase arrest site: 2
- DNA polymerase b frameshift hotspot 1: 1
- DNA polymerase a/b frameshift hotspot 1: 1
- DNA polymerase a/b frameshift hotspot 2: 1
- Ig heavy chain class switch repeat 1: 1
- Ig heavy chain class switch repeat 3: 1
- Ig heavy chain class switch repeat 4: 1
- Ig heavy chain class switch repeat 5: 1

**Suppl. Figure 8: Integrated epigenomic profile of the *RAX2* locus in adult human retina.** Alignment of profiles obtained by ATAC-seq and multiple ChIP-seq (H3K4me2, H3K27ac, Crx, Otx2) experiments, revealing genetic regions potentially involved in *cis*-regulation of *RAX2*. Coordinates of these regions, the *RAX2* gene, and its promoter are given below the figure. Data provided by Timothy J. Cherry (Harvard University, Cambridge, MA, United States). ROI, region of interest; CRE, *cis*-regulatory element. Deletion g.3771337_3774298del overlaps with the *RAX2* promoter and deletion g.3765788_3772920del overlaps with CRE_3 and CRE_4.

**
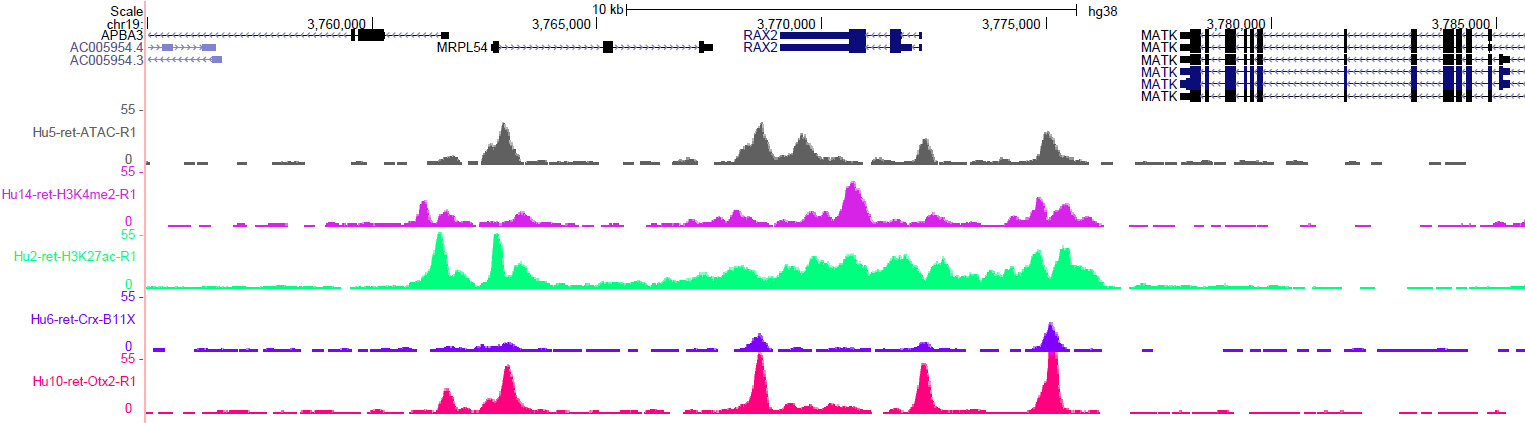
**

| **ROI** | **coordinates (hg19)** |
| --- | --- |
| CRE_1 | chr19:3,761,509-3,762,008 |
| CRE_2 | chr19:3,762,818-3,763,317 |
| CRE_3 | chr19:3,768,356-3,768,855 |
| CRE_4 | chr19:3,769,369-3,769,868 |
| **RAX2** | **chr19:3,769,089-3,772,219** |
| promoter | chr19:3,772,047-3,772,546 |
| CRE_5 | chr19:3,774,791-3,775,290 |

**REFERENCES**

1. Abeysinghe SS, Chuzhanova N, Krawczak M, et al. (2003) Translocation and gross deletion breakpoints in human inherited disease and cancer I: Nucleotide composition and recombination-associated motifs. *Hum Mutat* **22** 229-244.
2. Conrad DF, Pinto D, Redon R, et al. (2010) Origins and functional impact of copy number variation in the human genome. *Nature* **464** 704-712.
3. Plagnol V, Curtis J, Epstein M, et al. (2012) A robust model for read count data in exome sequencing experiments and implications for copy number variant calling. *Bioinformatics* **28** 2747-2754.
4. Rice P, Longden I, Bleasby A. (2000) EMBOSS: the European Molecular Biology Open Software Suite. *Trends Genet* **16** 276-277.
5. Verdin H, D’haene B, Beysen D, et al. (2013) Microhomology-mediated mechanisms underlie non‑recurrent disease-causing microdeletions of the FOXL2 gene or its regulatory domain. *PLOS Genet* **9** e1003358.
